# Genome-wide association and multi-trait analyses characterize the common genetic architecture of heart failure

**DOI:** 10.1038/s41467-022-34216-6

**Published:** 2022-11-14

**Authors:** Michael G. Levin, Noah L. Tsao, Pankhuri Singhal, Chang Liu, Ha My T. Vy, Ishan Paranjpe, Joshua D. Backman, Tiffany R. Bellomo, William P. Bone, Kiran J. Biddinger, Qin Hui, Ozan Dikilitas, Benjamin A. Satterfield, Yifan Yang, Michael P. Morley, Yuki Bradford, Megan Burke, Nosheen Reza, Brian Charest, Regeneron Genetics Center, Renae L. Judy, Megan J. Puckelwartz, Hakon Hakonarson, Atlas Khan, Leah C. Kottyan, Iftikhar Kullo, Yuan Luo, Elizabeth M. McNally, Laura J. Rasmussen-Torvik, Sharlene M. Day, Ron Do, Lawrence S. Phillips, Patrick T. Ellinor, Girish N. Nadkarni, Marylyn D. Ritchie, Zoltan Arany, Thomas P. Cappola, Kenneth B. Margulies, Krishna G. Aragam, Christopher M. Haggerty, Jacob Joseph, Yan V. Sun, Benjamin F. Voight, Scott M. Damrauer

**Affiliations:** 1 Division of Cardiovascular Medicine, Perelman School of Medicine, University of Pennsylvania, Philadelphia, PA USA; 2 Corporal Michael J. Crescenz VA Medical Center, Philadelphia, PA USA; 3 Department of Surgery, University of Pennsylvania Perelman School of Medicine, Philadelphia, PA USA; 4 Department of Genetics, University of Pennsylvania Perelman School of Medicine, Philadelphia, PA USA; 5 Department of Epidemiology, Rollins School of Public Health, Emory University, Atlanta, GA USA; 6 The Charles Bronfman Institute of Personalized Medicine, Icahn School of Medicine at Mount Sinai, New York, NY USA; 7 Department of Medicine, Stanford University School of Medicine, Stanford, CA USA; 8 Regeneron Genetics Center, Tarrytown, NY USA; 9 Genomics and Computational Biology Graduate Group, Perelman School of Medicine, University of Pennsylvania, Philadelphia, PA USA; 10 Center for Genomic Medicine, Massachusetts General Hospital, Harvard Medical School, Boston, MA USA; 11 Program in Medical and Population Genetics and Cardiovascular Disease Initiative, Broad Institute of MIT and Harvard, Cambridge, MA USA; 12 Cardiovascular Research Center, Massachusetts General Hospital, Boston, MA USA; 13 Emory University School of Public Health, Atlanta, GA USA; 14 Atlanta VA Health Care System, Decatur, GA USA; 15 Departments of Internal Medicine and Cardiovascular Medicine, and Mayo Clinician-Investigator Training Program, Mayo Clinic, Rochester, MN USA; 16 Department of Cardiovascular Medicine, Mayo Clinic, Rochester, MN USA; 17 Cardiovascular Institute, Perelman School of Medicine, University of Pennsylvania, Philadelphia, PA USA; 18 Massachusetts Veterans Epidemiology Research and Information Center, VA Boston Healthcare System, Boston, MA USA; 19 Department of Pharmacology, Center for Genetic Medicine, Northwestern University Feinberg School of Medicine, Chicago, IL USA; 20 Center for Applied Genomics, The Children’s Hospital of Philadelphia, Philadelphia, PA USA; 21 Division of Nephrology, Department of Medicine, Vagelos College of Physicians & Surgeons, Columbia University, New York, NY USA; 22 Department of Pediatrics, Division of Human Genetics and Center for Autoimmune Genomics and Etiology, Cincinnati Children’s Hospital Medical Center, Cincinnati, OH USA; 23 Department of Preventive Medicine, Feinberg School of Medicine, Northwestern University, Chicago, IL USA; 24 Center for Genetic Medicine, Bluhm Cardiovascular Institute, Northwestern University Feinberg School of Medicine, Chicago, IL USA; 25 Department of Preventive Medicine, Northwestern University Feinberg School of Medicine, Chicago, IL USA; 26 The Charles Bronfman Institute for Personalized Medicine, BioMe Phenomics Center, and Department of Genetics and Genomic Sciences, Icahn School of Medicine at Mount Sinai, New York, NY USA; 27 Division of Endocrinology, Emory University School of Medicine, Atlanta, GA USA; 28 Cardiovascular Research Center and Cardiac Arrhythmia Service, Massachusetts General Hospital, Boston, MA USA; 29 Division of Nephrology, Department of Medicine, Icahn School of Medicine at Mount Sinai, New York, NY USA; 30 Institute for Biomedical Informatics, University of Pennsylvania Perelman School of Medicine, Philadelphia, PA USA; 31 Department of Translational Data Science and Informatics and Heart Institute, Geisinger, Danville, PA USA; 32 Department of Medicine, Brigham and Women’s Hospital, Harvard Medical School, Boston, MA USA; 33 Department of Systems Pharmacology and Translational Therapeutics, University of Pennsylvania Perelman School of Medicine, Philadelphia, PA USA; 34 Institute of Translational Medicine and Therapeutics, University of Pennsylvania Perelman School of Medicine, Philadelphia, PA USA

**Keywords:** Genetic association study, Genomics, Cardiology, Cardiomyopathies, Heart failure

## Abstract

Heart failure is a leading cause of cardiovascular morbidity and mortality. However, the contribution of common genetic variation to heart failure risk has not been fully elucidated, particularly in comparison to other common cardiometabolic traits. We report a multi-ancestry genome-wide association study meta-analysis of all-cause heart failure including up to 115,150 cases and 1,550,331 controls of diverse genetic ancestry, identifying 47 risk loci. We also perform multivariate genome-wide association studies that integrate heart failure with related cardiac magnetic resonance imaging endophenotypes, identifying 61 risk loci. Gene-prioritization analyses including colocalization and transcriptome-wide association studies identify known and previously unreported candidate cardiomyopathy genes and cellular processes, which we validate in gene-expression profiling of failing and healthy human hearts. Colocalization, gene expression profiling, and Mendelian randomization provide convergent evidence for the roles of *BCKDHA* and circulating branch-chain amino acids in heart failure and cardiac structure. Finally, proteome-wide Mendelian randomization identifies 9 circulating proteins associated with heart failure or quantitative imaging traits. These analyses highlight similarities and differences among heart failure and associated cardiovascular imaging endophenotypes, implicate common genetic variation in the pathogenesis of heart failure, and identify circulating proteins that may represent cardiomyopathy treatment targets.

## Abstract (web summary)

Heart failure is a major cause of cardiovascular morbidity and mortality. Here, the authors report results of a genome-wide association study meta-analysis, characterizing the role of common genetic variants in heart failure, finding overlap with common cardiovascular risk factors and imaging measures of cardiac structure/function.

## Introduction

Heart failure (HF) is a common cardiovascular syndrome characterized by symptoms including shortness of breath, volume-overload, and functional limitation that result from structural or functional impairment of ventricular filling or ejection of blood^1–4^. HF affects >38 million individuals globally, with rapidly growing prevalence, and is a major cause of cardiovascular morbidity, mortality, hospitalization, and healthcare costs^5^. Despite the prevalence of HF, the role of common genetic variation in HF risk remains poorly understood. In comparison to other common cardiometabolic traits like coronary artery disease (CAD), myocardial infarction, diabetes, blood pressure, and obesity, where hundreds of genetic loci have been associated with disease risk, discovery of common genetic sequence variants associated with HF has been modest, with only 11 genomic loci identified in the largest genome-wide association study (GWAS) of HF to date^6^.

Several strategies have been described to improve power for GWAS locus discovery. One approach, particularly useful for improving the generalizability of GWAS findings, is to improve the genetic diversity of participants included in the study. Simulation and empiric studies have identified power gains of multi-ancestry GWAS^7^, recently demonstrated with applications to CAD and blood lipids^8,9^. Refining clinical phenotypes is another effective approach to improve detection of common variant associations. This approach may be particularly relevant, as HF is a heterogeneous clinical syndrome^10^. For example, in prior work parsing dilated cardiomyopathy from all-cause HF using diagnosis and procedure codes facilitated the identification of common-genetic variation in genes associated with Mendelian forms of cardiomyopathy^11^. More recently, statistical methods that jointly consider related phenotypes have been developed. These multi-trait GWAS methods leverage the shared genetic relationships between related traits, and have been shown to improve power for genetic discovery across a range of diseases^12–14^. For example, a recent multi-trait analysis jointly considered hypertrophic cardiomyopathy, dilated cardiomyopathy, and cardiac imaging traits, successfully identifying novel common genetic variants associated with these traits^15^. Because structural cardiac abnormalities and dysfunction of the left ventricle are key contributors to the development of clinical HF, and these traits share genetic architecture, we hypothesized that jointly considering HF and related continuous quantitative cardiac imaging phenotypes within a multi-trait GWAS framework may similarly improve power for genetic discovery.

In this study, we report a multi-ancestry meta-analysis of HF genome-wide association studies to estimate the effect of common genetic sequence variants on all-cause HF risk. We integrate GWAS of cardiac imaging endophenotypes and HF using multivariate GWAS methods to further improve power for locus discovery. Finally, we evaluate the genetic evidence for these associations using colocalization, transcriptome-wide association, gene-expression profiling, and Mendelian randomization. In summary, this study identifies previously unreported HF risk variants and putative effector genes, prioritizes relevant tissues, highlights roles for common genetic sequence variation in the pathogenesis of HF and related traits, and identifies circulating proteins associated with HF and cardiovascular imaging phenotypes.

## Results

### Multi-ancestry HF meta-analysis identifies 47 risk loci

**Fig. 1:**
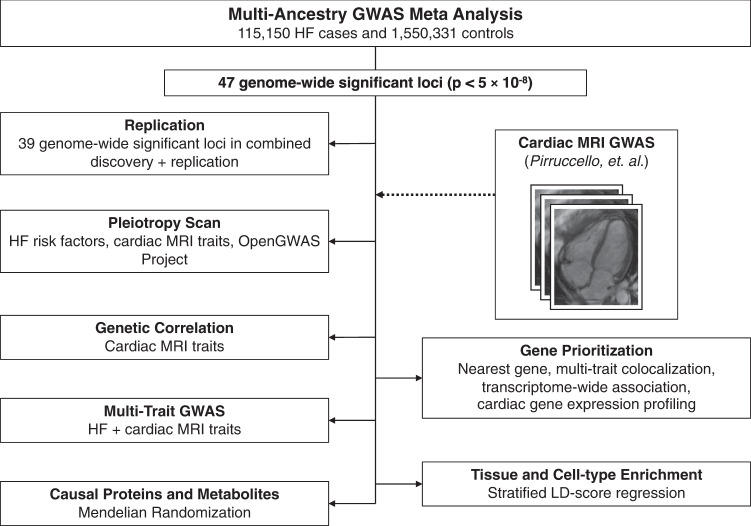
Study overview. Overview of major study analyses to identify HF-associated genetic variants, shared risk loci with other traits/diseases, prioritize genes/tissues/cell types, and identify potential treatment targets. GWAS genome-wide association study, HF heart failure, LD linkage disequilibrium.

**Fig. 2:**
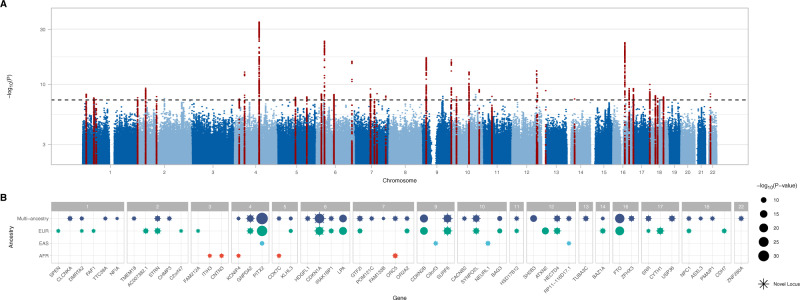
Genome-wide associations for heart failure. Results of the multi-ancestry GWAS meta-analysis of all-cause heart failure, performed using a fixed-effect inverse variance weighted model. **A** Manhattan plot of genome-wide significant (*p* < 5 × 10^−8^) associations. Each point represents a genetic variant. Variants in red are located +/−500 kb of a genome-wide significant locus. The *x*-axis represents the genomic position, and the *y*-axis represents the strength of association as represented by −log_10_(*p* value). **B** Candidate genes were assigned to each genome-wide significant variant (*p* < 5 × 10^−8^) in the multi-ancestry and ancestry-specific analyses (based on proximity to the nearest transcription start site). Candidate genes are grouped by chromosome. Previously unreported candidate genes (>500 kb from a previously reported locus) are denoted by stars. The size of each point corresponds to the strength of association as represented by −log_10_(*p* value). Where multiple independent variants mapped to the same gene, only the strongest association is shown.

The overall study design is presented in Fig. 1. We conducted a multi-ancestry GWAS meta-analysis of all-cause HF, including 115,150 HF cases and 1,550,331 controls of diverse genetic ancestry, assembled from large consortia and medical biobanks (HERMES, Penn Medicine Biobank, eMERGE, Mount Sinai BioMe, Geisinger DiscovEHR, FinnGen, and the Global Biobank Meta-analysis Initiative)^6,16–19^ (Supplementary Data 1 and Supplemental Fig. 1). In the discovery phase, we identified 939 variants at 47 loci where genetic associations reached the genome-wide significance (GWS) threshold (*p* < 5 × 10^−8^) (Fig. 2A and Supplementary Data 2). Of the 12 independent variants previously reported from the HERMES consortium GWAS of all-cause HF^6^, 10 remained genome-wide significant in the current analysis (*p* < 5 × 10^−8^), and the remaining two were significant at a Bonferroni-adjusted threshold (*p* < 0.05/12). Of the 47 loci we identified, 34 were located >500 kb from the lead variants reported by the largest published GWAS of all-cause HF^6^. Consistent with prior reports^6^, the strongest association with HF was found at the PITX2 locus (Fig. 2B). There was nominal evidence of heterogeneity at *PITX2*, *LPA*, *CDKN2B*, and *RP11-116D17.1* loci by genetic ancestry, but these effects were not significant after accounting for multiple testing (0.05/47 < *I*^2^ *p* value <0.05) (Supplementary Data 2).

We sought replication in data from the VA Million Veteran Program (43,344 HF cases, and 258,943 controls of European Ancestry)^20^ and Mass General Brigham Biobank (5,542 HF cases and 20,242 controls of European Ancestry). Of 47 genome-wide significant risk loci in our analysis, 44 were available for replication (Supplementary Data 3). Of these 44 loci, 41 (93%) had concordant direction of effect (exact binomial *p* = 8.1 × 10^−10^), and 27 were associated with HF at a Bonferroni-adjusted significance threshold (*p* < 0.05/44 = 0.001). In all, 37/44 loci were associated with HF at a nominal significance threshold (*p* < 0.05) with concordant direction of effect. In a combined discovery + replication meta-analysis, 39/44 loci reached the genome-wide significance threshold (*p* < 5 × 10^−8^).

### Pleiotropy scan reveals shared associations with cardiometabolic traits

**Fig. 3:**
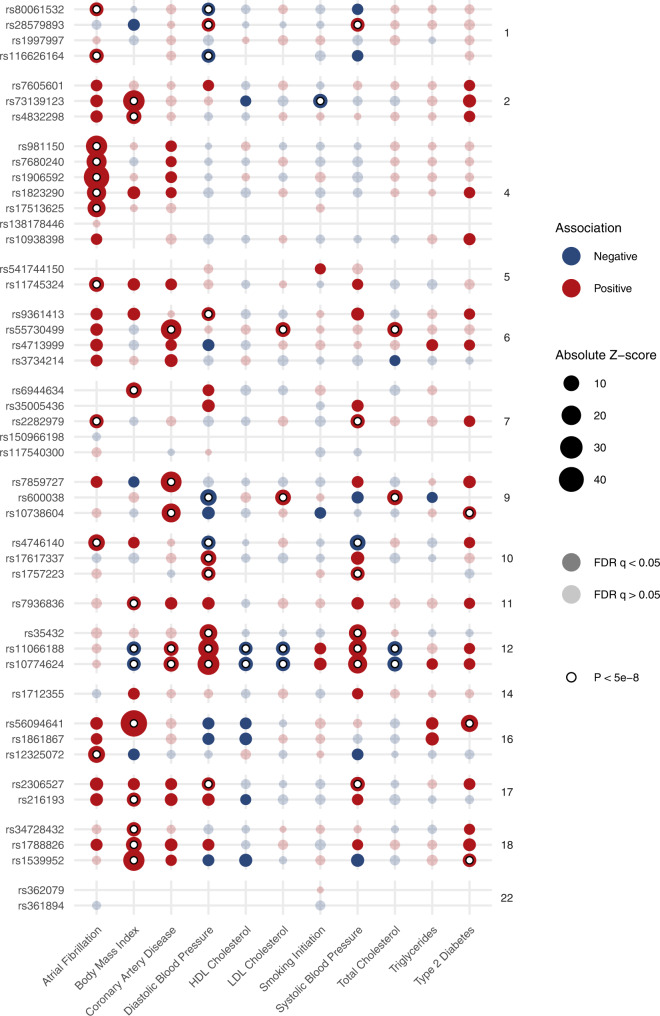
Associations of heart failure risk variants with common cardiometabolic traits. Dotplot indicating associations between lead variants at heart failure risk loci (*y*-axis), with common cardiometabolic traits (*x*-axis), with summary estimates obtained from GWAS reported in the IEU OpenGWAS Project. Of the 47 lead risk variant for HF, 46 (or a proxy) were reported in at least 1 cardiometabolic trait GWAS. The size of each point denotes the absolute z-score for each trait, with reference to the heart-failure increasing allele. The shading of each point denotes whether the association met an FDR adjustment for multiple testing. Associations exceeding the conventional genome-wide significance threshold are denoted with a white circle. Variants are grouped by chromosome. FDR false discovery rate.

To determine associations between the HF loci and other traits, we queried summary-level results from 34,513 GWAS collected by the MRC-IEU OpenGWAS Project (https://gwas.mrcieu.ac.uk/). The lead variants at the novel HF loci shared pleiotropic associations with a wide variety of traits (Supplementary Data 4), including known and common HF risk factors. Of 47 genome-wide significant loci, 32 were associated with at least 1 common cardiometabolic trait (Atrial Fibrillation, Body Mass Index, Coronary Artery Disease, Diastolic Blood Pressure, HDL Cholesterol, LDL Cholesterol, Smoking Initiation, Systolic Blood Pressure, Total Cholesterol, or Type 2 Diabetes) at genome wide significance (Fig. 3 and Supplementary Data 5). The most common shared associations occurred with diastolic blood pressure (12 loci), atrial fibrillation (11 loci), body mass index (11 loci), systolic blood pressure (8 loci), and coronary artery disease (5 loci). Several loci shared associations with multiple cardiometabolic traits, including rs10774624 near *SH2B3* (7 cardiometabolic traits) and rs11066188 near *HECTD4* (7 traits). Although the HF-increasing alleles at these loci are associated with favorable markers of metabolic health including lower body mass index, total-, and LDL-cholesterol, these loci are also associated with increased blood pressure and coronary artery disease, which may explain the association with increased HF-risk.

### HF and cardiac structure/function phenotypes are genetically correlated

**Fig. 4:**
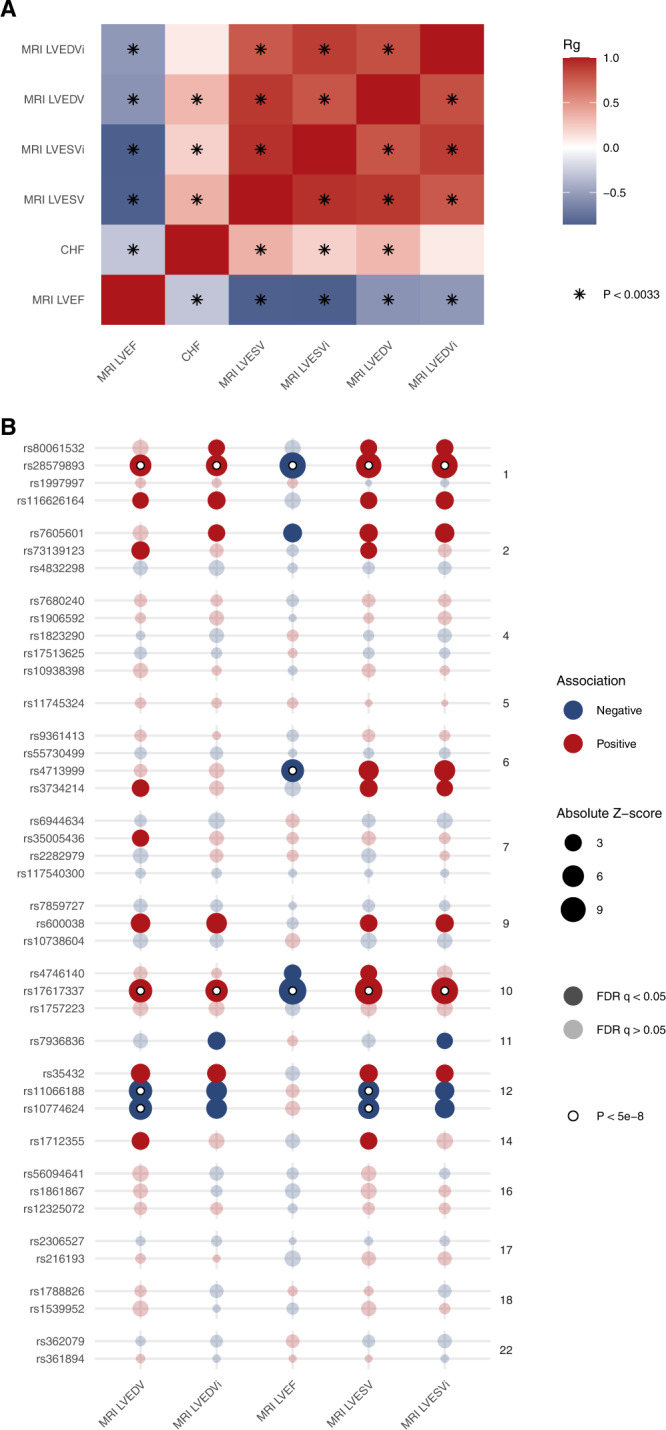
Shared associations between heart failure and cardiac MRI traits. **A** Cross-trait LD score regression was performed to estimate genetic correlations (*r*_g_) between heart failure and cardiac MRI traits. Significant associations using the Bonferroni method to account for multiple testing are noted with a star. **B** GWAS associations between lead heart failure risk variants and cardiac MRI traits. The size of each point denotes the absolute z-score for each trait, with reference to the heart failure increasing allele. The shading of each point denotes whether the association met an FDR adjustment for multiple testing. Associations exceeding the conventional genome-wide significance threshold are denoted with a white circle. Variants are grouped by chromosome. LVEDV left-ventricular end-diastolic volume, LVEDVi left-ventricular end-diastolic volume indexed for body surface area, LVSEV left-ventricular end-systolic volume, LVESVi left-ventricular end-systolic volume indexed for body surface area, LVEF left-ventricular ejection fraction, FDR false discovery rate.

Clinically, HF is diagnosed in the setting of typical symptoms occurring in the presence of abnormalities of cardiac structure/function^4^. Cross-trait linkage disequilibrium score regression (LDSC)^21,22^ was performed to estimate the genetic correlation of HF with previously-reported cardiac imaging-derived measures of cardiac structure and function including: cardiac MRI-derived measures of left-ventricular end-diastolic volume (LVEDV_MRI_), left-ventricular end-systolic volume (LVESV_MRI_), volumes indexed for body surface area (LVEDVi_MRI_ and LVESVi_MRI_), and left-ventricular ejection fraction (LVEF_MRI_) obtained from a GWAS of cardiac MRI traits among 36,041 healthy UK Biobank participants^23^. HF was significantly correlated with all imaging endophenotypes except LVEDVi_MRI_, with the strongest correlation between HF and LVESV_MRI_ (*r*_g_ = 0.36; *p* = 3.73 × 10^−^^16^; Fig. 4A and Supplementary Data 6).

Five common (MAF > 0.01) HF lead risk variants were associated with a cardiac MRI parameter at a genome-wide level of significance, and 10 additional loci were associated with MRI measures at a more liberal FDR *q* < 0.05 (Fig. 4B). While most HF-associated variants were associated with reduced ejection fraction and larger left ventricular volumes, rs10774624 near *SH2B3* and rs11066188 near *HECTD4* were associated with smaller left ventricular volumes, potentially indicative of a HF with preserved ejection fraction phenotype.

### Multivariate genome-wide analysis of HF endophenotypes identifies novel loci

Having established significant genetic correlations and shared risk variants between HF and cardiac imaging phenotypes suggestive of common genetic etiology, we applied multi-trait GWAS methods (N-GWAMA, multi-trait analysis of genome-wide association summary statistics (MTAG), and a common factor model specified within the Genomic Structural Equation Modeling framework) to improve the power to discover additional associated genetic variants^12–14^. These methods make different assumptions about the shared relationships and heritability among the input traits, but are robust to scenarios where SNP-trait associations are derived from overlapping samples, and have been previously been demonstrated to improve power for genetic discovery^12–14^. This analysis utilized the results of the multi-ancestry HF meta-analysis performed above, along with the previously-published cardiac MRI GWAS^23^. Given the stronger genetic correlations and larger number of shared risk loci between HF and cardiac MRI measures that were not indexed for body surface area, we focused our primary analysis on HF, LVEF, LVEDV_MRI_, and LVESV_MRI_. We additionally considered MRI measures indexed for body surface area in a sensitivity analysis.

**Fig. 5:**
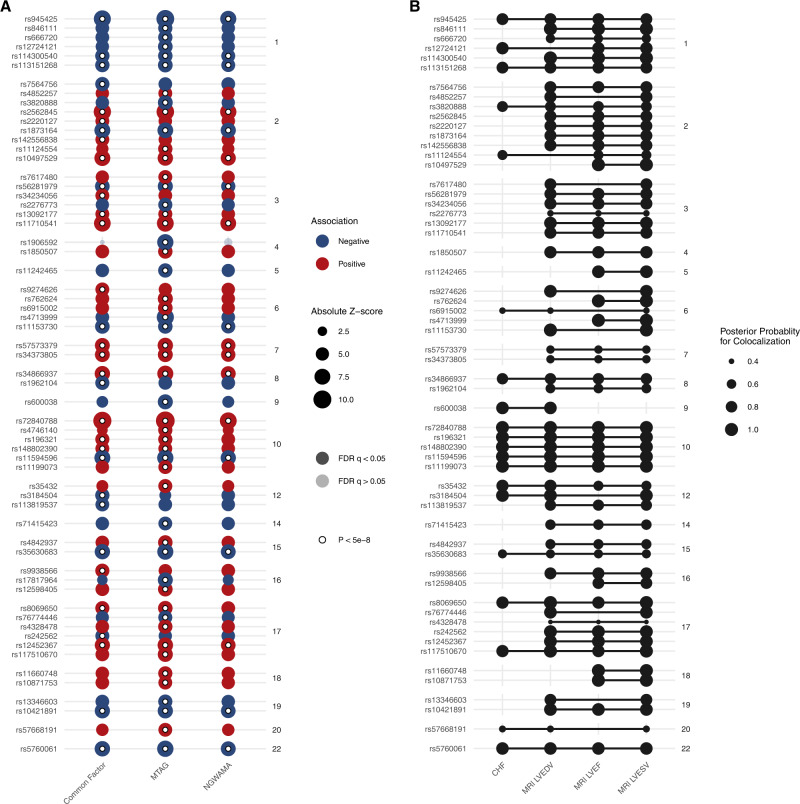
Results of multivariate genome wide association study. Multivariate GWAS and multi-trait colocalization were performed to identify genetic loci associated with HF and cardiac structure/function traits. **A** Results of multivariate GWAS. The *x*-axis denotes the multivariate GWAS method, and the *y*-axis denotes the independent lead variants at each locus. The size of each point denotes the absolute *z*-score for each trait. The shading of each point denotes whether the association met an FDR adjustment for multiple testing. Associations exceeding the conventional genome-wide significance threshold are denoted with a white circle. Variants are grouped by chromosome. **B** Results of multi-trait colocalization. The *x*-axis denotes heart failure and cardiac imaging traits. The *y*-axis represents the lead variant at each independent locus identified in the multivariate GWAS. Lines connect groups of traits with evidence of colocalization at a given locus. The size of each point represents the posterior probability for colocalization. Evidence for colocalization was determined based on the default variant specific regional and alignment priors $({P}_{{{{{{\rm{R}}}}}}}^{*}={P}_{{{{{{\rm{A}}}}}}}^{*}=0.5)$, with colocalization identified when ${P}_{{{{{{\rm{R}}}}}}}{P}_{{{{{{\rm{A}}}}}}}\ge 0.25$. FDR false discovery rate.

Across the three multi-trait methods, we identified 61 independent loci (Fig. 5A and Supplementary Data 7). Of the 61 lead variants at these loci, 14 did not reach genome-wide significance in any of the parent studies. Overall, lead variants at 9 of the 61 GWS loci were located nearest to known cardiomyopathy genes (*ACTN2*, *ALPK3*, *BAG3*, *FLNC*, *PLN*, *TTN*), representing significant enrichment (hypergeometric *p* = 6.01 × 10^−^^11^). In a sensitivity analysis considering HF, LVEF, LVEDV and LVESV indexed for body surface area, or in a combined analysis including both indexed and unindexed ventricular volumes, we similarly identified significant enrichment of loci located near known cardiomyopathy genes (BSA-indexed: *ACTN2*, *ALPK3*, *BAG3*, *FLNC*, *TTN*, hypergeometric *p* = 6.82 × 10^−^^4^; combined: *ACTN2, ALPK3, BAG3, FLNC, PLN, TTN*, hypergeometric *p* = 1.28 × 10^−^^6^). Genome-wide significant variants from these analyses are reported in Supplementary Data 8, and Manhattan plots are reported in Supplemental Figs. 4–6.

To further corroborate the relevance of these loci across HF and imaging traits, we performed multi-trait colocalization^24^. This method simultaneously evaluates the probability of a shared causal variant at a locus across multiple traits. We found evidence for colocalization across two or more HF/imaging traits at 58 of the 61 loci, suggesting the multivariate GWAMA results represent discovery of shared genetic etiologies among the input traits (Fig. 5B and Supplementary Data 9).

Among the 14 novel loci prioritized in the multi-trait analyses were several which have been previously linked to other cardiovascular traits/diseases in GWAS or functional studies. Among these loci include rs846111 encoding a missense variant in the *RNF207* gene, which encodes a heart-specific ring-finger protein linked to cardiac energy homeostasis^25^. This variant has been previously associated with increased QT interval^26^. We identified a strong novel association at rs4328478, an intronic variant located within the *PRKCA* gene on chromosome 17. Functional studies of rs9912468, a *cis-*eQTL for *PRKCA* in near-perfect linkage disequilibrium with rs4328478 (EUR *r*^2^ = 0.996), previously identified an association in this region with expression of *PRKCA* in the human left ventricle, with zebrafish and in vitro reporter assays suggestive of cardiac-specific enhancer activity at this locus^27^. In humans, the *PRKCA* locus has previously been associated with electrocardiographic measures of left ventricular mass at genome-wide significance^27^, and nominally associated with echocardiographic traits and dilated cardiomyopathy^27^, now reaching genome-wide significance for HF. We detected another association at rs6915002, an intronic variant near *MLIP* that encodes the Muscular LMNA Interacting Protein. This highly-conserved gene has been established as a key cardiac sensitizer to stress, regulating morphologic adaptation (hypertrophy and dilation) in a series of murine overexpression and deletion experiments^28,29^. Another strong novel association was rs3820888, an intronic variant located near the *SPATS2L* gene, which has previously been implicated in atrial fibrillation and QT-interval^30^. Many of the novel loci in the multivariate analysis have been previously associated with known HF risk factors including measures of blood pressure and diabetes (hemoglobin A1c), markers of arrhythmias (e.g., atrial fibrillation, QT-interval, pulse rate), blood cell traits, as well as anthropometric traits such as height and body fat/mass-related traits (Supplementary Data 10).

Additional loci uniquely prioritized when considering MRI measures indexed for body surface area included intronic variants located near *MITF, FAF1*, and *TCF7L2*, among others (Supplementary Data 8 and Supplemental Figs. 4–6). *MITF* (rs1430608) is expressed in the heart, and has previously been linked to beta-adrenergic-induced cardiac hypertrophy in mice and congenital heart disease in humans^31,32^. *FAF1* (rs12096443) encodes a member of the Fas death-induced signaling complex, and contributes to stress-induced apoptosis of cardiomyocytes^33^. While *TCF7L2* is an important risk locus for diabetes^34^, the specific variant we identified (rs34943800) has not been previously linked to diabetes. In human, rat, and murine HF, *TCF7L2* has been identified as a binding partner of β-catenin, acting to mediate Wnt signaling leading to cardiac hypertrophy^35^.

### Tissue and cell-type enrichment

To determine whether genetic associations for HF were enriched for specific tissues or cell-types we applied LDSC-SEG, a form of stratified of LD score regression which partitions heritability among sets of specifically expressed genes^36^. We detected significant associations (*p* < 0.05) with gene expression (GTEx) and chromatin marks (ROADMAP and ENTEX) in several tissues. The strongest association with gene expression was in the left ventricle (*p* = 8.5 × 10^−4^), and the strongest association with chromatin marks was with H3K36me3 in psoas muscle (Supplemental Fig. 7 and Supplementary Data 11). We leveraged single nucleus RNA sequencing (snRNA-seq) data from MAGNet to identify associations with cardiac-specific cell types, finding enrichment with cardiomyocytes (Supplemental Fig. 7)^37^. Many cardiometabolic traits are known to influence risk of HF, and we detected nominal enrichment of signals within adipose, blood, and endocrine tissues.

### Colocalization, transcriptome-wide association, and gene-expression profiling analyses prioritize HF effector genes

To prioritize putative candidate genes associated with HF risk, we sought several lines of evidence. As GWAS loci are frequently not located within protein-coding locations of the genome, mapping these GWAS variants to genes and pathways is important for functional interpretation. In addition to mapping variants to the nearest gene, we applied colocalization, transcriptome-wide association, gene expression profiling, and Mendelian randomization. Results of these analyses are described below, and a summary of genes prioritized across multiple methods is presented in Supplemental Fig. 9.

#### Multi-trait colocalization

Colocalization is a method which can be used to integrate gene expression data with GWAS results to enable mapping of GWAS variants to genes^38^. We performed multi-trait colocalization to identify shared genetic signals associated with gene expression in the heart, HF, and MRI traits, as studying related traits simultaneously increases power to detect putative causal variants^24^. We utilized an expression quantitative trait loci (eQTL) dataset from the Myocardial Applied Genomics Network (MAGNet), derived from 313 human hearts (177 failing hearts, 136 donor nonfailing control hearts)^39^. In total, genetic loci linked to expression of 32 genes colocalized with GWAS signals for HF and/or cardiac imaging traits (Supplementary Data 12).

The genes with strongest evidence for colocalization included *DNAJC18*, *MTSS1*, *SQLE*, *BCKDHA*, *ABO*, *ALPK3*, and *PROM1*. These genes have been previously linked to HF and other cardiovascular traits: For example, epigenetic marks at *DNAJC18* have been linked to dilated cardiomyopathy^40^; *MTSS1* has been linked in candidate-variant studies with HF traits, with knockout in mice associated with changes in echocardiographic measures of HF^41^; epigenetic marks at *SQLE* have been previously linked to HF with preserved ejection fraction^42^; *BCKDHA* (which encodes branched chain keto acid dehydrogenase E1 subunit alpha, a key enzyme responsible for branch chain amino acid (BCAA) degradation) has been implicated in adverse cardiac remodeling and HF^43–45^; *ABO* has been linked to myocardial infarction;^46^ *ALPK3* is an established cardiomyopathy gene associated with both dilated and hypertrophic cardiomyopathies^47,48^; and *PROM1* is a marker of fibroblast progenitor cells that has been previously linked to cardiac fibrosis^49^.

We found strong evidence for colocalization between *BCKDHA* expression in healthy hearts, failing hearts, LVEDV_MRI_ and LVESV_MRI_ (posterior probability 0.98). The heart is a major source of BCAA catabolism^50^, and BCAA have been linked to HF phenotypes in several model systems^43–45^. *BCKDHA* expression was also increased in failing compared to healthy hearts (EUR fold change = 1.25, *p* = 0.005; AFR fold change = 1.24, *p* = 0.043). Given the colocalization evidence for a shared genetic signal influencing *BCKDHA* expression and LVEDV_MRI_ and LVESV_MRI_, we performed Mendelian randomization to determine whether circulating branch-chain amino acid (isoleucine, leucine, valine) levels may be causally associated with LVEDV_MRI_ and LVESV_MRI_. Using genetic instruments derived from a GWAS of circulating metabolites among up to 24,925 participants of ten European studies^51^, Increased circulating levels of isoleucine and leucine were significantly associated with decreased LVEDV_MRI_ (leucine *β* = −0.137, 95% CI −0.25 to −0.022, *p* = 0.02; isoleucine *β* = −0.276, 95% CI −0.38 to −0.17, *p* = 3 × 10^−7^) and LVSEV_MRI_ (leucine *β* = −0.131, 95% CI −0.24 to −0.026, *p* = 0.01; isoleucine *β* = −0.217, 95% CI −0.33 to −0.11, *p* = 1 × 10^−4^), with no significant associations identified for valine (Supplemental Fig. 8). We did not detect evidence of reverse-causality (e.g., increased LVEDV_MRI_ or LVESV_MRI_ leading to increased BCAA levels), and the findings remained robust when using the weighted-median MR method, which makes different assumptions about the presence of pleiotropy (Supplemental Fig. 8).

#### Transcriptome-wide association study (TWAS)

**Fig. 6:**
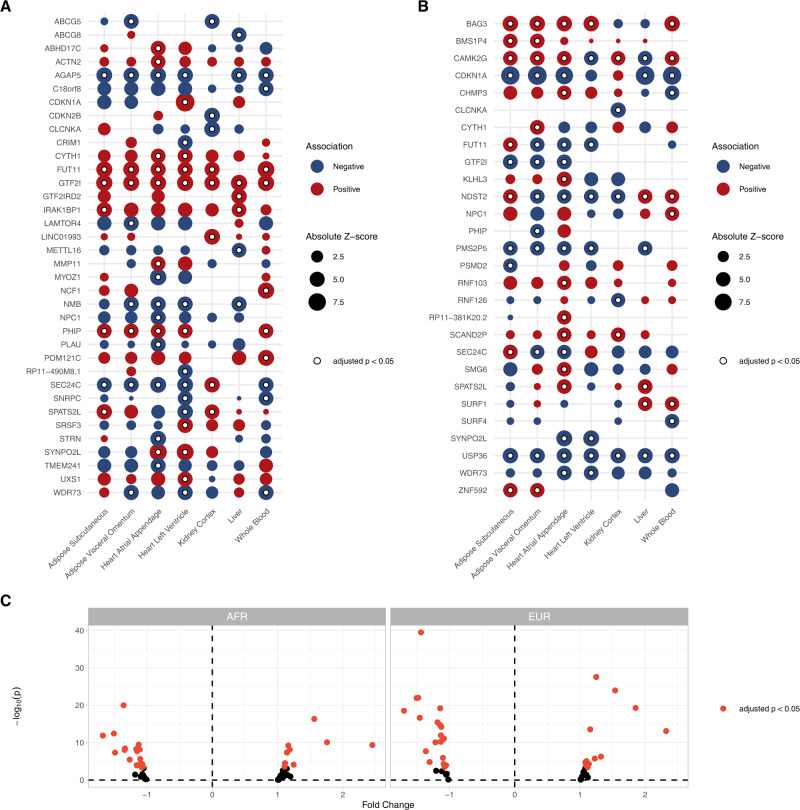
TWAS results. TWAS identified 36 distinct genes (representing 73 gene–tissue pairs) where expression was associated with adverse HF/structure/function traits, and 28 distinct genes (across 111 splicing–tissue pairs) where splicing was associated with adverse HF/structure/function traits. **A** Dotplot depicting the gene–tissue pairs where gene expression was significantly associated with HF. **B** Dotplot depicting the gene–tissue pairs where transcript splicing was significantly associated with HF. In **A**, **B**, the bubble size corresponds to absolute *z*-score, with bubbles colored to the direction of effect, while white dots denote associations that were significant after Bonferroni adjustment for multiple testing (*p* < 0.05/17703 genes). Only the most significant gene–tissue pair is shown when multiple splicing events in a given gene were identified. **C** Left ventricular gene expression profiling from MAGNet for genes prioritized by TWAS. Red dots represent candidate genes with significant differential expression among failing vs. healthy hearts, after Bonferroni adjustment for multiple testing.

Next, we performed TWASs integrating gene expression and splicing data from the Genotype-Tissue Expression (GTEx) project with the results of our HF GWAS^52–55^. We performed TWAS using models from cardiometabolic tissues (left ventricle, atrial appendage, visceral adipose, subcutaneous adipose, liver, kidney, and blood), to identify genes where tissue-specific expression levels (eQTL) or transcript splicing events (sQTL) may be relevant to HF. Across all tissues, we identified 36 distinct genes representing 73 gene-tissue pairs where gene expression was significantly associated with HF after Bonferroni adjustment for multiple testing (79,965 gene–tissue pairs) (Fig. 6A and Supplementary Data 13). We also identified 111 splicing events across 28 genes that were significantly associated with HF after Bonferroni adjustment for multiple testing (187,456 splicing event-tissue pairs) (Fig. 6B and Supplementary Data 14). The set of genes identified by the eQTL and sQTL TWAS was enriched for Mendelian cardiomyopathy genes (*BAG3, ACTN2)* (hypergeometric *p* = 0.049).

Among the most highly prioritized TWAS associations was *CLCNKA* gene expression in kidney (*p* = 1.51 × 10^−19^ in eQTL and *p* = 6.53 × 10^−9^ in sQTL analyses). *CLCNKA* encodes the *K*_a_ renal chloride channel (ClC-*K*_a_), with a prior candidate-variant study identifying a suggestive association between the common coding variant rs10927887 and HF^56^. Further functional characterization of this variant revealed loss-of-function in the ClC-*K*_a_ chloride channel, implicating a Bartter syndrome-like cardio-renal axis in HF^56^. Although other genes in this region including *HSPB7*^57^ and *ZBTB17*^58^ have been implicated in HF, our results provide support for a role of *CLCNKA*. Other highly prioritized genes included *CDKN1A* (*p* = 7.56 × 10^−17^ in the atrial appendage), an important cell-cycle regulator of cardiomyocyte proliferation during terminal differentiation;^59,60^ *SYNPO2L* (*p* = 9.15 × 10^−13^ in the atrial appendage and *p* = 9.64 × 10^−12^ in the left ventricle), a Z-disc protein previously linked to atrial fibrillation^61,62^.

#### Gene expression profiling

To validate the TWAS findings we compared expression levels of genes prioritized by the TWAS analyses (Bonferroni *p* < 0.05) among 166 healthy (122 EUR, 44 AFR) and 166 failing (89 EUR, 77 AFR) hearts from MAGNet. Of 49 genes with available expression data, we identified 34 genes where expression significantly differed between healthy and failing hearts after Bonferroni adjustment for multiple testing (*p* < 0.05 adjusting for 49 genes) (Fig. 6C and Supplementary Data 15).

#### Gene ontology

We performed an exploratory gene ontology analysis^63^ among the TWAS-prioritized genes. Cellular Component analysis was notable for enrichment of several sarcomere components including the Z-disc, I-band, sarcomere overall, and myofibrils. (Supplementary Data 16). Enriched biological processes included cholesterol metabolism (*ABCG5*, *ABCG8*, *NPC1*), and muscle cell development/assembly/organization (*ACTN2*, *SYNPO2L*, *MYOZ1*) (Supplementary Data 17).

### Proteome-wide Mendelian randomization prioritizes circulating proteins associated with adverse HF phenotypes

**Fig. 7:**
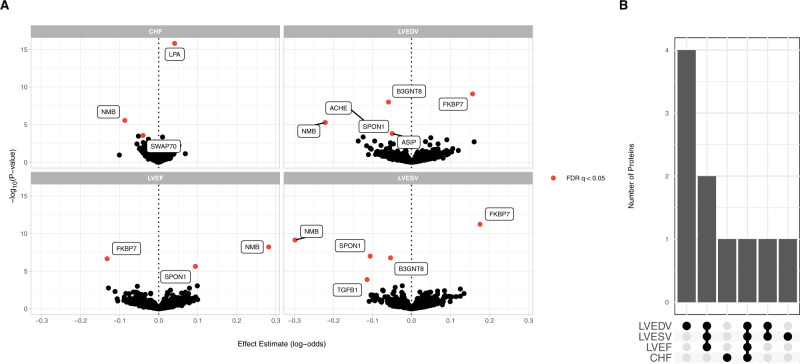
Proteome-wide Mendelian randomization. Proteome-wide MR was performed using high-confidence genetic instruments to detect associations between circulating proteins and cardiac endophenotypes. **A** Associations between circulating protein levels and HF traits estimated using Mendelian randomization. Protein-trait associations passing an FDR (false discovery rate) *q* < 0.05 are highlighted. **B** Number of shared associations across HF traits.

Finally, we performed an unbiased proteome-wide Mendelian randomization analysis using high-confidence genetic instruments for 725 circulating proteins to identify their contribution to each of the HF endophenotypes. We identified 17 significant (FDR < 0.05) protein–trait associations, across 9 distinct circulating proteins (Fig. 7A and Supplementary Data 18). Among these significant protein-trait pairs was lipoprotein(a) (*LPA*), with increasing levels associated with increased risk of HF (OR 1.04 per 1−SD increase in lipoprotein(a), 95% CI 1.03 to 1.05, *p* = 1.6 × 10^−16^). Lipoprotein(a) is a known risk factor for coronary artery disease that has been previously associated in observational studies with incident HF and associated hospitalization^64,65^. To further evaluate this association, we considered an additional genetic instrument previously reported to explain >40% of the variation in circulating Lp(a) levels across multiple cohorts^66^. Using this genetic instrument, each 10 mg/dL increase in Lp(a) was associated with a small but significant increased risk of HF (OR 1.014, 95% CI 1.01 to 1.02, *p* = 5.7 × 10^−22^). This association was no longer significant in multivariable MR accounting for the association of these genetic variants with coronary artery disease (OR 1.01, 95% CI 0.99 to 1.02, *p* = 0.53), indicating CAD may mediate the effects of lipoprotein(a) in the pathogenesis of HF. These findings are consistent with prior identification of *LPA* as a HF GWAS locus^6^, and with observational findings which suggest the effects of Lp(a) on incident HF are likely attributable to coronary artery disease^67^.

We additionally identified four circulating proteins that were associated with multiple traits (Fig. 7B). *NMB* was associated with increased LVEF, decreased ventricular volumes, and decreased risk of HF. Other proteins associated with multiple traits included SPON1, FKBP7, and B3GNT8. (Fig. 7A). Circulating SPON1 and FKBP7 have both been previously linked to atrial fibrillation, a known HF risk factor^68^, and SPON1 has been identified as a potential biomarker for HF hospitalization^69^.

## Discussion

In this study, we performed multi-ancestry and multi-trait genome-wide meta-analyses of HF and related cardiac imaging traits. We analyzed the genetic relationships among these traits (1) demonstrating improved power for novel locus discovery for this collection of traits, (2) implicating both known and previously unreported variants in HF pathogenesis, and (3) prioritizing genes, pathways, and circulating proteins for future study in the pathogenesis of HF.

First, these findings highlight the value of multi-ancestry and multi-trait genome wide analyses to improve genetic discovery. Although GWAS of HF have been performed largely in populations of European ancestry, HF is a global disease associated with high morbidity and mortality. Consistent with GWAS of other cardiovascular traits like CAD^8^, we found that multi-ancestry analysis improved power for discovery, identifying 47 HF loci (compared with 11 in the largest previously-published analysis). Similarly, incorporating HF endophenotypes in multi-trait analyses further improved power for discovery. Across both analyses, we identified many variants which had previously been associated with cardiometabolic and anthropometric traits, which are themselves HF risk factors. With the growth of institutional biobanks that link rich electronic health records, laboratory, and imaging findings with genetic data, more refined phenotyping efforts (including studies focused on variation within the normal range among otherwise healthy individuals) may further enable genetic discovery within this multi-trait paradigm.

Importantly, our findings highlight the value of integrating large-scale GWAS of common, complex traits with smaller, more focused studies of specific quantitative endophenotypes. GWAS of many cardiovascular endophenotypes have been previously reported, including among largely healthy populations. A common finding across these studies has been the observation that the genes and pathways identified by studying variation in cardiovascular traits among largely disease-free populations recapitulate known disease-associated genes and pathways. For example, recent GWAS of thoracic aorta diameters among healthy individuals implicate known Mendelian aortopathy genes like *FBN1*^70,71^, GWAS of electrocardiographic traits find long-QT syndrome-associated genes like *SCN5A* are associated with variation in many other subclinical electrocardiographic traits^72^, and GWAS of both left- and right-heart structure/function among healthy individuals identify Mendelian cardiomyopathy genes^23,73,74^. We demonstrate an example of linking GWAS of a common, complex trait like HF, with GWAS of detailed cardiac structure/function endophenotypes, and recapitulate known Mendelian cardiomyopathy genes and sarcomere components. Incorporating detailed phenotyping of biomarkers and imaging traits among smaller, healthy cohorts, particularly of diverse genetic ancestry, may be an important opportunity for further improve genetic discovery.

Second, our findings implicate known and previously unreported HF effector genes and pathways. We found convergent evidence for the influence of common variants across several secondary analyses, summarized in Supplemental Fig. 9. For example, the gene supported across the most lines of evidence was *STRN*. This gene encodes striatin, a calmodulin binding protein which has been linked to the intercalated disc of cardiac myocytes, colocalizes with desmosomal proteins, and associates with spontaneous arrhythmogenic cardiomyopathy in canines^75^. Other highly prioritized genes across multiple lines of evidence include Mendelian cardiomyopathy genes (*BAG3, ACTN2, ALPK3*). We also detected significant enrichment of genes associated with cellular contractile machinery and cardiomyocyte development and structure. Overall, these findings highlight the utility of integrative analyses that draw on genetic association, gene expression, chromatin modification, and tissue/cell-type-specific datasets to prioritize disease-associated genes. These findings suggest that while HF has pleiotropic contributors, similar genes, tissues, and cellular components associated with Mendelian forms of HF may be important for common manifestations as well.

Although our findings implicate some genes and pathways with putative links to cardiomyocyte structure/function, other loci appear to have pleiotropic effects, and are linked to HF risk factors like atrial fibrillation or blood pressure. HF is a heterogeneous disease, varying in etiology, severity, and age of onset, among other factors. As we studied a broad “all-cause” definition of HF, these findings are not surprising. Future studies of more homogeneous HF definitions may help clarify the relevance of specific pathways to HF subtypes, help resolve pleiotropy, and clarify whether some variants may be associated with diseases that phenocopy HF-like chronic lung diseases and obesity. Prior studies have also broadly suggested that polygenic risk may modify the impact of rare but more severely damaging variants^15,76^. Future work investigating the interactions between common and rare HF variants, as well as genetic determinants of common HF risk factors may be useful in clarifying the pathways that determine the heterogeneous clinical presentations.

Finally, we observed potentially causal links between circulating metabolites and proteins with HF and related imaging traits. Elevated circulating levels of branch chain amino acids have been previously implicated as a risk factor for incident HF and adverse cardiac remodeling in a murine model of myocardial infarction^45,77^. Our colocalization and MR analyses identified strong evidence of a shared genetic etiology and potentially causal relationship between circulating leucine and isoleucine levels and left ventricular volumes, although the pathologic relevance to human HF specifically requires further investigation.

### Limitations

This study has several possible limitations. First, although we performed the largest multi-ancestry GWAS of HF to-date, these analyses included a large number of participants of European ancestry. We were able to replicate the majority of our genetic associations, but replication was limited to cohorts of European ancestry. As the global burden of HF is increasing, future GWAS of HF and related traits (particularly quantitative imaging traits) in other diverse populations is warranted. Future multi-ancestry analyses will hopefully further improve our understanding of the true breadth of the common genetic basis of these traits. In downstream colocalization, TWAS, and gene expression profiling analyses we included multi-ancestry cohorts (GTEx v8, MAGNet), which should overall improve generalizability of our findings. Second, the definition of HF in many cohorts was based on diagnosis codes, which may have resulted in phenotype misclassification. We included highly correlated imaging traits with biologically plausible connections to HF to maximize interpretability of our multi-trait analysis and enhance identification of cardiac-specific loci. Including other cardiovascular and HF endophenotypes (e.g., circulating biomarkers like natriuretic peptides) may further improve discovery. Efforts to develop specific HF phenotyping definitions^11^, and statistical methods to account for misclassification^78^ may also be helpful in ensuring GWAS of HF identify bona fide risk loci, rather than variants that may be primarily associated with phenocopies like obesity and chronic lung disease. Third, these results represent the findings of a selection of multi-trait GWAS methods which make different assumptions about disease heritability and genetic relatedness. No gold-standard multi-trait GWAS method exists, and whether locus discovery or biologic relevance may be further improved with other methods requires further study. Similarly, no gold-standard gene prioritization framework exits. We applied several methods which provided biologically plausible candidate genes supported by prior literature; however, other bioinformatic or functional approaches may prioritize different candidate genes. Functional assessment of HF-associated risk variants, genes, and pathways in model systems will be important to validate their role in HF. Finally, although incidence of HF is similar among men and women, epidemiologic differences have been noted, and sex-specific subsets of HF exist (e.g., peripartum cardiomyopathy)^79^. Here, we did not perform sex-stratified analyses, or analyses focused specifically on identifying associations on the sex chromosomes. Whether sex-specific genetic associations exist will be an important area of future study.

In summary, these analyses highlight similarities and differences among HF and associated cardiovascular imaging endophenotypes, implicate common genetic variation in the pathogenesis of HF, and identify circulating proteins and metabolites that may represent cardiomyopathy treatment targets.

## Methods

### Genome wide association study meta-analysis

In the discovery phase, GWAS summary statistics for HF were obtained from non-overlapping analyses of six separate cohorts/consortia (HERMES, Penn Medicine Biobank, eMERGE, Mount Sinai BioMe, Geisinger DiscovEHR, FinnGen, and the Global Biobank Meta-analysis Initiative)^6,16–19^. All-cause HF was defined using cohort-specific definitions (pheCodes^80^ or ICD9/10 codes documented within the electronic health record for all studies except HERMES, which additionally included expert adjudication among some cohorts) (Supplemental Methods). Details of study-specific genotyping and quality control are available in the Supplemental Methods, and included standard local controls for missingness, sex discordance, variant-level factors including missingness, Hardy–Weinberg equilibrium, and imputation accuracy. Analyses were performed separately by cohort and ancestry, adjusted for age, sex, and population structure. Prior to meta-analysis, GWASinspector^81^ was used to perform study-level quality control using default settings to evaluate for test statistic inflation, skewness, kurtosis, allele frequency mismatches, and perform allele harmonization. Fixed-effects meta-analysis was performed using METAL^82^ using the inverse-variance weighted (standard error) method within and across ancestries to generate ancestry-specific and multi-ancestry meta-analysis summary statistics. Fine-grained ancestry estimation from summary statistics was performed using *bigsnpr*^83,84^. Independent significant genomic risk loci were defined using the “–clump” command in PLINK^85^ and the 1000 Genomes Phase 3 reference panel (*p* < 5 × 10^−8^; window 500 kb; linkage disequilibrium *r*^2^ = 0.6, *r*_2_^2^ = 0.1). Lead variants (or proxies identified using LDlinkR^86^) at each independent risk locus were carried forward for replication. For replication, fixed effects meta-analysis was performed to combine data from the VA Million Veteran Program and Mass General Brigham Biobank (Supplemental Methods). Discovery + replication phase data for each independent risk locus was combined in a final fixed effects meta-analysis. All genomic positions are reported using coordinates from the GRCh37 build of the human genome.

### Genetic correlation of HF and cardiac imaging traits

Cross-trait linkage-disequilibrium score regression (LDSC) was performed to estimate genetic correlation (*r*_g_) between HF, and cardiac MRI traits (LVEF, LVEDV, LVESV, LVEDVi, LVESVi; UKB http://kp4cd.org/datasets/mi)^21,22^. LDSC is a computationally efficient method which utilizes GWAS summary statistics to estimate heritability and genetic correlation between polygenic traits while accounting for sample overlap.

### Multivariate GWAS

Genome-wide association study summary statistics were obtained for HF and cardiac MRI (LVEF, LVEDV, LVESV, LVEDVi, LVESVi) traits. The primary analysis included HF and LVEF, LVEDV, LVESV, while MRI measures indexed for body surface area were included in a sensitivity analysis. Variants were filtered to include common (MAF > 0.01) variants present in the 1000 Genomes Phase 3 reference panel. Genomic Structural Equation Modeling (SEM) is a framework which uses GWAS summary statistics to model the genetic covariance structure of complex traits. Genomic-SEM leverages a multivariate extension of cross-trait linkage-disequilibrium score regression (LDSC) to estimate genetic correlation (*r*_g_), quantify heritability, estimate dependence between traits, and account for up to complete sample overlap^13,21,22^. This approach is flexible, allowing the user to use systems of equations to model proposed relationships between the observed traits and latent variables. To estimate SNP-level effects, the genetic covariance and sampling covariance matrices (estimated using LD score regression) are expanded to include SNPs, which are then individually regressed on parameters specified by each structural model. We specified a common factor model, as well as models corresponding to MTAG and N-weighted multivariate GWAMA frameworks^12–14^. Details of model specifications are available in the Supplemental Methods. All multi-trait analyses were implemented using the *GenomicSEM* package in R using the diagonally-weighted least squares estimator^13^.

### Cardiomyopathy gene enrichment

We utilized a previously published list of Mendelian cardiomyopathy-associated genes^23^ aggregated from commercially-available gene panels to test for enrichment of genome-wide significant loci/genes. In sum, these panels contained 108 autosomal genes. To test enrichment, we applied the hypergeometric test in *R* to evaluate whether the set of candidate genes is over-represented in the set of established cardiomyopathy genes.

### Multi-trait colocalization

Statistical colocalization is a method to assess shared genetic etiology between traits. We used HyPrColoc^24^, a recently developed Bayesian algorithm designed to simultaneously and efficiently evaluate for colocalization across multiple traits using summary statistics. We assessed for colocalization across HF, MRI, and heart gene expression traits in the 500 kb region centered on the lead variants identified in the multivariate GWAS analyses. Gene expression data was derived from an expression quantitative trait loci (eQTL) dataset from the Myocardial Applied Genomics Network (MAGNet), derived from 313 human hearts (177 failing hearts, 136 donor nonfailing control hearts) obtained at time of organ procurement (control hearts) or heart transplant (failing hearts)^39^. Evidence for colocalization was determined based on the default variant specific regional and alignment priors $({P}_{{{{{{\rm{R}}}}}}}^{*}={P}_{{{{{{\rm{A}}}}}}}^{*}=0.5)$, with colocalization identified when ${P}_{{{{{{\rm{R}}}}}}}{P}_{{{{{{\rm{A}}}}}}}\ge 0.25$.

### Tissue and cell-type enrichment

We implemented LDSC-SEG^36^ to test for enrichment of disease heritability by integrating our GWAMA summary statistics with gene expression^87,88^, chromatin^89,90^, and cardiac-specific cell-type^37^ datasets. We applied false discovery rate correction separately across each dataset (gene expression, chromatin, and cardiac cell-type) to account for multiple testing, with FDR < 0.05 considered significant.

### Branched chain amino acid Mendelian randomization

Genetic variants associated with branch chain amino acids (leucine, isoleucine, and valine) at genome wide significance (*p* < 5 × 10^−8^) were identified from a previously reported GWAS meta-analysis including up to 24,925 participants of ten European studies^51^ Participants underwent genotyping and NMR profiling of circulating metabolites, and GWAS was performed to understand the contribution of common genetic variation to circulating metabolite levels. Genetic instruments were constructed from independent (EUR *r*^2^ < 0.3, distance = 10,000 kb) variants associated with each BCAA at genome-wide significance. The corresponding SNP effects were identified in GWAS summary statistics for LVEDV_MRI_ and LVSEV_MRI_, harmonized to consistent effect alleles, and two-sample inverse variance weighted Mendelian randomization with random effects was performed using the *TwoSampleMR* package in R^91^. Sensitivity analysis was performed using the weighted median method, which remains robust when up to 50% of the weight of the genetic instrument is invalid^92^.

### Transcriptome-wide association study

S-PrediXcan was used to integrate gene expression and splicing data from GTEx version 8 and GWAS summary statistics from the HF GWAS to identify genes associated with all-cause HF^52–55^. Pretrained gene expression and transcript splicing models from cardiometabolic tissues (left ventricle [*n* = 386 genotyped GTEx v8 samples], atrial appendage, [*n* = 372] visceral adipose [*n* = 469], subcutaneous adipose [*n* = 581], liver [*n* = 208], and kidney [*n* = 73]) were obtained from http://predictdb.org/. Bonferroni adjustment was performed to account for multiple testing (79,965 gene–tissue pairs for eQTL TWAS; 187,456 splicing event–tissue pairs for sQTL TWAS), with adjusted *p* < 0.05 considered significant. We tested for enrichment of TWAS-prioritized genes among Mendelian cardiomyopathy-associated genes using the hypergeometric distribution to yield a one-tailed *p* value.

### Cardiac gene expression profiling

The Myocardial Applied Genetics Consortium (MAGnet) is a multicenter, institutional review board-approved consortium designed to explore the genetic underpinnings of cardiac gene expression^39,93^. Briefly, human cardiac samples were obtained from failing hearts collected at time of heart transplantation, and from healthy donor hearts that were suitable for transplant but logistically did not reach recipients. RNA gene expression profiling on cardiac tissue samples obtained from the left ventricle was performed as previously described^39^. Gene expression profiling was performed using Affymetrix expression arrays, log-transformed, normalized to reference probes, batch normalized, and adjusted for gender, age, and collection site. Gene expression data was queried to determine whether significant expression differences existed between healthy and failing hearts for genes prioritized by TWAS, with significant fold-changes differences determined by Bonferroni-adjusted *p* < 0.05 to account for multiple testing.

### Biological pathway and cellular component analysis

Biological pathway and cellular component analysis was performed using ShinyGO, an online platform for gene enrichment analysis^63^. Based on a set of input genes, the application tests for enrichment among prespecified gene sets based on the hypergeometric distribution followed by false discovery rate correction for multiple testing. Pathways or components with FDR *q* < 0.05 were considered significant.

### Proteome-wide Mendelian randomization

Proteome-wide Mendelian randomization was performed as previously described (http://www.epigraphdb.org/pqtl/)^94^. Briefly, we identified high-confidence (tier 1) *cis*-acting genetic instruments for 725 circulating proteins, which passed previously defined consistency and pleiotropy tests and had available corresponding SNP effects from our HF meta-analysis and/or the cardiac MRI GWAS. When multiple SNPs were available for an exposure–outcome pair, inverse-variance weighted MR was performed as the primary analysis, with Wald-ratio MR performed when only one SNP was available for the exposure-outcome pair. FDR correction was applied to account for multiple testing, with *q* < 0.05 considered significant. For the Mendelian Randomization analysis of lipoprotein(a), a secondary genetic instrument was constructed using genetic variants previously reported to explain >40% of the variation in circulating Lp(a) levels. Of 43 previously reported genetic variants, 27 were available in our HF GWAS. Inverse-variance weighted MR was performed as the primary analysis, with MR-Egger and weighted median methods applied as sensitivity analyses, as these make different assumptions about the presence of invalid instruments and pleiotropy^95^. Multivariable MR was performed to evaluate whether the effects of Lp(a) on were attenuated by the effects of Lp(a) on coronary artery disease. Summary data on coronary artery disease was obtained from ref. 96. All Mendelian randomization analyses were performed using the TwoSampleMR package in R^91^.

All statistical analyses were performed using R version 4.0.3 (R Foundation for Statistical Computing, Vienna, Austria).

### Ethical approval

The UK Biobank obtained IRB approval from the North West Multi-centre Research Ethics Committee (approval number: 11/NW/0382), and participants provided informed consent. The BioBank Japan Project was approved by the research ethics committees at the Institute of Medical Science, the University of Tokyo, the RIKEN Yokohama Institute, and cooperating hospitals; participants gave written informed consent. FinnGen participants provided informed consent for biobank research, and the Coordinating Ethics Committee of the Hospital District of Helsinki and Uusimaa (HUS) approved the FinnGen Study protocol No. HUS/990/2017. The Penn Medicine BioBank is approved by the University of Pennsylvania, and participants gave written informed consent.

### Reporting summary

Further information on research design is available in the Nature Research Reporting Summary linked to this article.

## Supplementary information

Supplementary Information

Description of Additional Supplementary Files

Supplementary Data 1-18

Reporting Summary

## Data Availability

The datasets generated as part of this study are available from the corresponding author upon reasonable request. The GWAS summary statistics for heart failure (HERMES: http://kp4cd.org/datasets/mi; GBMI: https://www.globalbiobankmeta.org/; FinnGen: https://r5.finngen.fi/pheno/I9_HEARTFAIL_ALLCAUSE), and cardiac MRI (http://kp4cd.org/datasets/mi) traits are publicly available. Cardiac eQTL and RNA expression/sequencing data were provided by the Myocardial Applied Genomics Network (MAGNet; https://www.med.upenn.edu/magnet/). The summary statistics for the GWAS of all-cause heart failure generated in this study have been deposited in the GWAS Catalog database under accession code GCST90162626. The summary statistics for the GWAS of all-cause heart failure and the multi-trait GWAS have also been deposited at Zenodo at 10.5281/zenodo.7181277.

## Code availability

Publicly available software was used to perform the analyses. Code is available from the corresponding author upon reasonable request.
