# Supplementary material for: Genome-wide association and multi-trait analyses characterize the common genetic architecture of heart failure
Source: Nat Commun. 2022 Nov 14;13:6914. doi: 10.1038/s41467-022-34216-6 (PMC9663424; doi:10.1038/s41467-022-34216-6)
Supplement: Supplementary file 1 — Supplementary Information [file 41467_2022_34216_MOESM1_ESM.pdf]

Multi-ancestry and Multivariate Genome-Wide Analysis Highlights the Role of Common Genetic Variation in Cardiac Structure, Function, and Heart Failure-related Traits

SUPPLEMENTAL MATERIAL

SUPPLEMENTAL METHODS 2

STUDY POPULATIONS, PHENOTYPING, GENOTYPING, AND QUALITY CONTROL 3
MULTI-TRAIT GWAS 6

SUPPLEMENTAL FIGURES 8

SUPPLEMENTAL FIGURE 1: DISTRIBUTION OF GENETIC ANCESTRY 9
SUPPLEMENTAL FIGURE 2: QQ-PLOT OF HF META-ANALYSIS 10
SUPPLEMENTAL FIGURE 3: MULTI-ANCESTRY HF GWAS REGIONAL ASSOCIATION PLOTS 11
SUPPLEMENTAL FIGURE 4: MANHATTAN PLOT OF NGWAMA OF HF AND CARDIAC MRI TRAITS 16
SUPPLEMENTAL FIGURE 5: MANHATTAN PLOT OF MTAG OF HF AND CARDIAC MRI TRAITS 17
SUPPLEMENTAL FIGURE 5: MANHATTAN PLOT OF COMMON FACTOR GWAS OF HF AND CARDIAC MRI TRAITS 18
SUPPLEMENTAL FIGURE 5: TISSUE AND CELL-TYPE ENRICHMENT 19
SUPPLEMENTAL FIGURE 6: BRANCH CHAIN AMINO ACID MENDELIAN RANDOMIZATION 20
SUPPLEMENTAL FIGURE 7: SUMMARY OF PRIORITIZED GENES 21

SUPPLEMENTAL AUTHORS 22

SUPPLEMENTAL REFERENCES 23

SUPPLEMENTAL METHODS

Study Populations, Phenotyping, Genotyping, and Quality Control

HERMES Consortium: Details of the HERMES Consortium have been previously described[1]. Briefly, the HERMES Consortium included participants of European ancestry from 26 separate cohorts or population-based studies including the UK Biobank. Heart failure was defined based on study-specific criteria, including diagnosis codes, discharge codes, death certificate codes, and expert clinician evaluation. Genotyping was performed using study-specific high density genotyping arrays, and quality controls included sample/variant call rate, Hardy-Weinberg equilibrium, and minor allele frequency, with additional study-specific quality controls. Imputation was performed to a variety of imputation panels including 1000 Genomes and Haplotype Reference Consortium. Genome wide association studies were adjusted for covariates including age, sex, and genetic principal components where available. Summary level quality control was performed using EasyQC, prior to fixed-effects meta-analysis using METAL[2].

Penn Medicine BioBank: The Penn Medicine Biobank is a longitudinal genomics and precision medicine study in which participants consent to linkage of genomic information and biospecimens to the electronic health record. All patients receiving care at Penn Medicine (Philadelphia, PA) are eligible for enrollment, and more than 65,000 participants are currently enrolled. Heart failure was defined based on diagnosis codes documented within the electronic health record. International Classification of Diseases version 9/10 codes were mapped to pheCodes[3], and individuals with codes 425 or 428 (including subcodes) were considered heart failure cases. Individuals without these codes were considered controls. Genotyping was performed using the Infinium Global Screening Array (GSA) (Illumina, Inc. San Diego, CA), and the current analysis included 43,623 participants with available genotype data. Pre-imputation quality control was performed using PLINK[4,5] to exclude low marker call rate (<95%), low sample call rate (<90%), or sex discordance between genotype and reported sex. Imputation to the TOPMed reference panel (97,256 samples and 308,107,085 variants) was performed using the TOPMed Imputation Server, with phasing performed using EAGLE and imputation performed using MINIMAC4[6]. Identity-by-descent with a Pi-hat threshold of 0.25 to account for relatives up-to first cousins was performed on the imputed genotype data, and one sample per related pair was removed. Ancestry-specific GWAS (European and African Ancestry) were performed using PLINK[4,5] among variants meeting the following quality thresholds: $r^2 > 0.3$; minor allele frequency > 0.001 or minor allele count > 20, adjusted for age, sex, and 5 ancestry-specific genetic principal components. LiftOver was used to map genome positions from hg38 to hg19/GRCh37[7].

eMERGE: The genome-wide association study (GWAS) was conducted using the electronic Medical Records and Genomics (eMERGE) Network dataset. The case/control cohort was comprised of individuals 18 years or older from the following non-pediatric eMERGE sites: Marshfield Clinic, Vanderbilt University Medical Center, Kaiser Permanente/University of Washington Medical Center, Columbia University, Mayo Clinic, Northwestern University, Geisinger Health System, Harvard (Partners Health Care), Icahn School of Medicine at Mount Sinai, and Meharry Medical College. Cases were defined as having an occurrence of one or more of the following International Classification of Diseases (ICD-9 or ICD-10) codes: I11.0, I13.0, I13.2, I25.5, I42.0, I42.5, I42.8, I42.9, I50.0, I50.1, I50.9, 425.4, 428.0, 428.1, 428.9,

402.01, 402.11, 402.91, 404.01, 404.11, 404.91, 404.03, 404.13, 404.93, 425.4, I50.0, I50.1, I50.2, I50.4, I50.9, or/and I50.82. Controls were defined as any individuals not having any of the aforementioned ICD codes. We conducted ancestry-specific analyses, creating separate case/control cohorts for individuals self-identifying as “Black” and “White” in the “Race” variable in the eMERGE dataset. There were a total of 7,208 controls and 2,607 cases in the African cohort (total = 9,815 individuals), and 48,714 controls and 14,065 cases in the European cohort (total = 62,779 individuals).

Genotyping and quality control of eMERGE has been previously reported[8]. Imputed genotype data with a minor allele frequency threshold of 0.01 to conduct the GWAS. Plink-v2.0[4,5] was used for all analysis. For the quality control of the genetic data we checked for robust sex concordance, a marker call rate of 0.01, and a sample call rate of 0.01. Related individuals were dropped using a pihat > 0.25 threshold. A Hardy-Weinberg equilibrium p-value threshold of 1e-9 was used. This produced a total of 13,275,706 SNPs tested in the African cohort and 7,654,263 SNPs tested in the European cohort. Logistic regression was run using covariates sex, age and the first 5 principal components (PCs). PCs were calculated from the ancestry-specific cohorts.

Mount Sinai BioMe: BioMe is an electronic health record-linked clinical-care biobank which includes more than 45,000 participants of diverse ancestry. Participants are recruited from the Mount Sinai healthcare system (New York, NY). The current analysis included individuals of European or African self-reported race/ancestry to avoid overlapping the Global Biobank Meta-analysis Initiative dataset which included Hispanic individuals from BioMe. Individuals with heart failure were identified using electronic health record diagnosis codes. Individuals were considered heart failure cases if they had evidence of the following codes: ICD10: I11.0, I13.0, I13.2, I25.5, I42.0, I42.5, I42.8, I42.9, I50.0, I50.1, I50.9; ICD9: 4254, 4280, 4281, 4289. Genotyping was performed using the Global Screening Array (Illumina, Inc. San Diego, CA). Genotype quality controls included checks for sex discordance, sample duplicates, low call rate (<95%), Hardy-Weinberg Equilibrium ($p < 1x10^{-5}$Individuals with closer than second-degree relatedness were removed using KING[9]. Genotypes were imputed using the TOPMed imputation server (https://imputation.biodatacatalyst.nhlbi.nih.gov). Ancestry-specific GWAS were performed using PLINK version 2 among well-imputed variants with minor allele frequency > 0.01, adjusting for age, sex and 10 genetic principal components[4,5]. LiftOver was used to map genome positions from hg38 to hg19[7].

Geisinger DiscovEHR: DiscovEHR is a collaboration between Geisinger and the Regeneron Genetics Center. The population is derived from patients who have previously consented to participate in the Geisinger MyCode Community Health Initiative. MyCode is an IRB-approved research study, and all participants have provided informed consent for broad use of samples for research. Participants are broadly recruited to MyCode from both primary and specialty care clinics across the Geisinger system. Exome sequencing and genome-wide genotyping of samples are performed by Regeneron, and genomic data are linked with Geisinger’s long-standing electronic health record, comprising both inpatient and outpatient records. This study included data from 82,608 patients who were genotyped on the Illumina Global Screening Array

chip out of 144,204 total patients in the cohort. Heart failure status was assigned based on ICD-10 codes. Details of genotyping and quality control have been previously reported[10]. The DiscovEHR participants included in the current analysis were distinct from participants included in the previously-published HERMES GWAS.

Global Biobank Meta-analysis Initiative: The Global Biobank Meta-analysis Initiative (GBMI) is a network of 19 biobanks representing >2 million consenting participants, with linkage of electronic health record and genotype data. Details of phenotyping, genotyping, quality control, and GWAS in GBMI have been reported previously[11]. To avoid overlap with other datasets included elsewhere in the current meta-analysis, we included two GWAS from GBMI (admixed-American and East Asian ancestry studies). Individuals were considered heart failure cases based on pheCode or ICD codes recorded within electronic health records, using study-specific definitions adapted from a GBMI-recommended definition. Genotyping was performed individually by biobank using genotype arrays. Following standard sample- and variant-level quality control genotypes were imputed into reference panels including 1000 Genomes, Haplotype Reference Consortium, or TOPMed. Ancestry-specific GWAS were performed by study, with suggested covariates including age, age^2, sex, age*sex, 20 first principal components, and biobank specific covariates including genotyping batches and recruiting centers. GWAS were recommended to be performed using SAIGE or REGENIE[12,13]. LiftOver was used to map genome positions from hg38 to hg19[7].

FinnGen: FinnGen is a public-private partnership aiming to collect genome and health data on 500,000 Finnish biobank participants. The study consists of ~200,000 legacy samples primarily collected by the National Institute for Health and Welfare, and an additional ~300,000 samples to be prospectively collected from hospital biobanks. Participating individuals consent to linkage of genome-wide genotyping with nationwide registers of longitudinal health data. Individuals with heart failure were identified using inpatient, outpatient, insurance reimbursement, and medication records, based on the “I9_HEARTFAIL_ALLCAUSE” phenotype (https://risteys.finngen.fi/phenocode/I9_HEARTFAIL_ALLCAUSE). Details of genotyping and quality control are available from https://finngen.gitbook.io/documentation/. Briefly, individuals underwent genotyping using Illumina or Affymetrix chip arrays. Individuals with ambiguous gender, high genotype missingness (>5%), excess heterozygosity (+/- 4 standard deviation), and non-Finnish ancestry were excluded. Variants with high-missingness, low Hardy-Weinberg Equilibrium p-value) $<1 \times 10^{-6}$), and minor allele count <3 were excluded. Samples were pre-phased using EAGLE[14], and imputed to the SISu v3 imputation reference panel using BEAGLE 4.1[15]. LiftOver was used to map genome positions from hg38 to hg19[7]. FinnGen participants provided informed consent for biobank research, and the Coordinating Ethics Committee of the Hospital District of Helsinki and Uusimaa (HUS) approved the FinnGen Study protocol No. HUS/990/2017.

VA Million Veteran Program (replication): Details of the VA Million Veteran Program (MVP) have been previously described[16,17]. Briefly MVP recruits participants from the Department of Veterans Affairs Healthcare System, who consent to linkage of electronic health records with biospecimens, surveys, and genomic information. More than 850,000 participants have

enrolled, with genomic and electronic health record data currently available for approximately 650,000. The current analysis focused on European Ancestry participants from MVP Release 3.0, which included 43,344 HF cases and 258,943 controls. HF phenotyping has been previously described, and was based on a combination of structured (ICD codes), and unstructured (ejection fraction) data extracted from the electronic health record[17]. Participants underwent genotyping using a custom Affymetrix Axiom Biobank Array[16]. Genotyping quality control has been previously described, and excluded duplicate samples, samples with more heterozygosity than expected, missing genotype calls (>2.5%), or discordance between genetically inferred sex and phenotypic gender[18]. One individual from each pair of related individuals (more than second degree relatedness as determined by KING[9]) was excluded. Variants were imputed to the 1000 Genomes Phase 3 version 5 reference panel using MINIMAC4[6]. Following imputation, low-quality (r^2<0.3) were excluded from further analysis. Ancestry was assigned using HARE as previously described[19]. PLINK2[4,5] was used to test for associations between each common (minor allele frequency >0.01) directly measured or imputed variant and all-cause heart failure, adjusted for age, sex, and ten genetic principal components.

Mass General Brigham Biobank (replication): The Mass General Brigham (formerly "Partners Healthcare") Biobank is a large research data and sample repository comprising more than 100,000 participants that is embedded within the framework of Mass General Brigham Personalized Medicine[20]. Participants are prospectively enrolled in the context of outpatient visits, inpatient stays, and emergency department encounters. The Biobank contains banked samples (plasma, serum, DNA and buffy coats), genomic data, and other health information, including data from the electronic health record (EHR) at hospitals affiliated with the Mass General Brigham Healthcare system – primarily the Massachusetts General Hospital and the Brigham and Women's Hospital in Boston, MA. Array- based genotyping was performed using either the Illumina Multi-Ethnic Genotyping Array, Expanded Multi-Ethnic Genotyping Array, or the Multi-Ethnic Global BeadChip Array (Illumina, Inc., San Diego, CA). We studied the first 25,784 genotyped participants of European genetic ancestry from the Mass General Brigham Biobank with relevant clinical data available. Individuals with heart failure were identified using electronic health record diagnosis codes for heart failure (ICD I50) OR cardiomyopathy (ICD I42) (at least one instance of either code). After standard genotype and sample quality control, imputation to the Haplotype Reference Consortium Version 1.1 reference panel was performed using the Michigan Imputation Server[6]. After imputation, SNPs were removed if missing rate < 0.02, Hardy-Weinberg Equilibrium p-value < 1e-06, or minor allele frequency < 1%, and samples were removed if missing rate > 0.05. PLINK2[4,5] was used to test for associations between each variant and all-cause heart failure, adjusted for age, sex, and five genetic principal components

Multi-trait GWAS
Multivariate models were implemented using the *GenomicSEM* package in R. We applied 3 models: a common factor model, and models analogous to MTAG and NGWAMA. Detailed description of the *GenomicSEM* package is available at: https://github.com/GenomicSEM/GenomicSEM. A detailed comparison of these approaches as implemented in *GenomicSEM* is presented in Grotzinger et. al[21]. Each method begins by fist

estimating the genetic covariance matrix using GWAS summary statistics and a multivariate extension of LD-score regression. Then each model is specified using a system of equations. Finally, the parameter(s) of interest are regressed on each SNP. A common factor model specifies a latent variable which represents the shared variance among related traits. This latent trait can variably influence each of the downstream traits (in this case HF and cardiac MRI measures of structure/function). A common factor GWAS thus considers the effects of genetic variants on the shared heritability of related traits. Here, the common factor GWAS considers a latent trait which influences HF and cardiac imaging measures of cardiac structure/function. MTAG was initially described by *Turley et. al.*[22], and is based on the concept that when traits are correlated, the precision of GWAS estimates can be improved when jointly modeling the correlated traits. The method was initially described as a generalized method of moments estimator and was subsequently adapted to the *GenomicSEM* framework in *Grotzinger et. al.*[23] Here, we specified a system of equations where 1) our target phenotype of interest (HF) was regressed on each SNP, and 2) the supporting phenotypes (in this case the cardiac MRI traits) were regressed on the target phenotype (HF). The results of this GWAS represent more precise SNP-effects on HF, and by virtue of increasing precision also improves power for novel discovery. Finally, the NGWAMA model was described by *Baselmans et. al.,*[24] and assumes each genetic variant has a common effect on each of the related traits. This method computes a multivariate Z-statistic which represents a weighted sum of test statistics from each of the input traits adjusted for sample overlap and genetic correlation between traits. An analogous model was specified in *GenomicSEM* using a common factor model where residual variances of each HF/MRI trait were fixed to 0, with the diagonally-weighted least squares estimator serving as an approximation of N-weighting.

SUPPLEMENTAL FIGURES

Supplemental Figure 1: Distribution of Genetic Ancestry

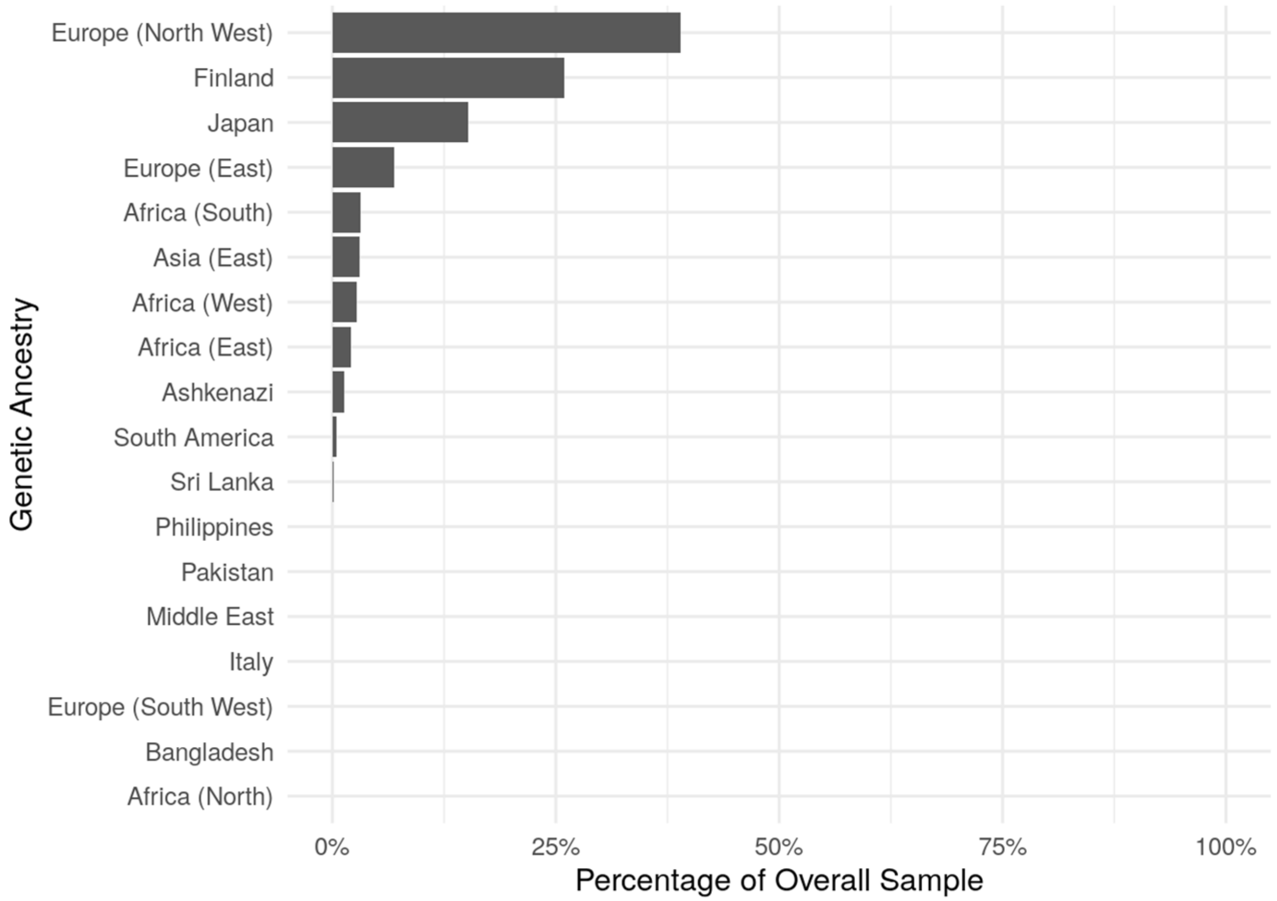

Distribution of genetic ancestry of included participants, as determined by projecting principal components of allele frequency into a reference sample of UK Biobank participants using the *snp_ancestry_summary()* function of the *bigsnpr* R package[25,26].

Supplemental Figure 2: QQ-plot of HF Meta-analysis

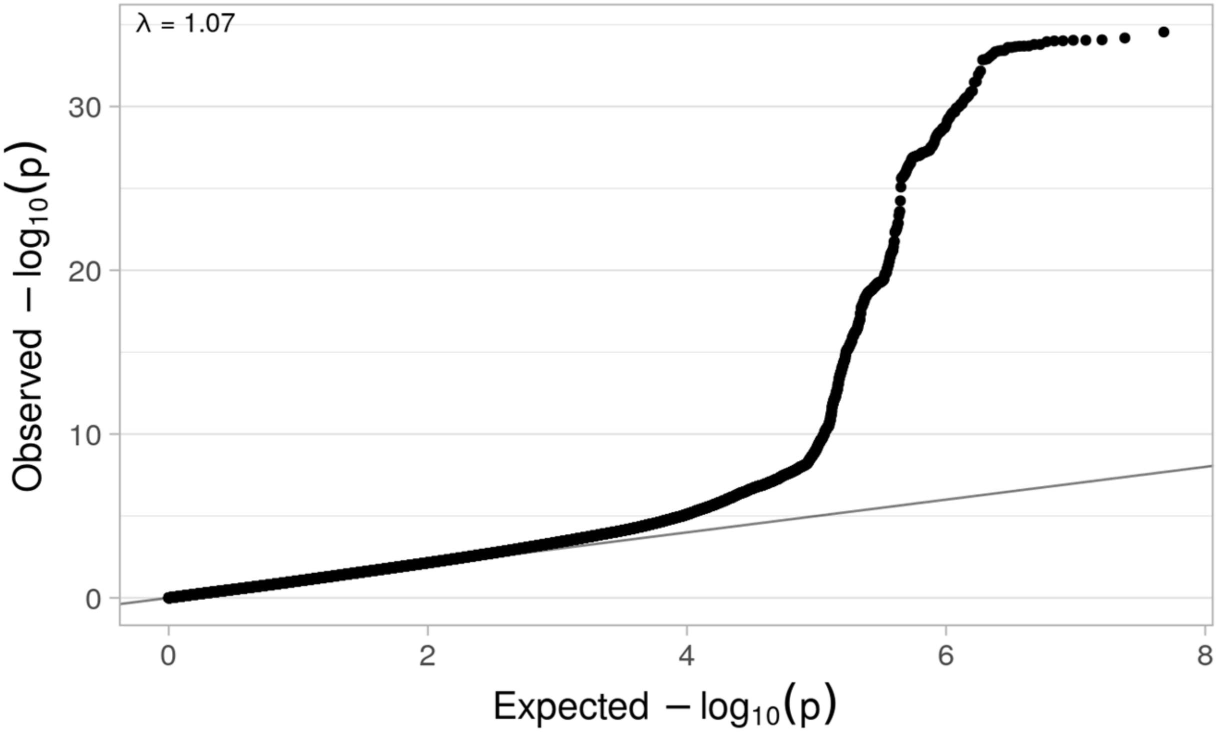

Quantile-quantile plot demonstrating the observed vs. expected p-value for variants included in the all-cause HF meta-analysis, estimated using fixed-effects inverse variance weighting.

Supplemental Figure 3: Multi-Ancestry HF GWAS Regional Association Plots

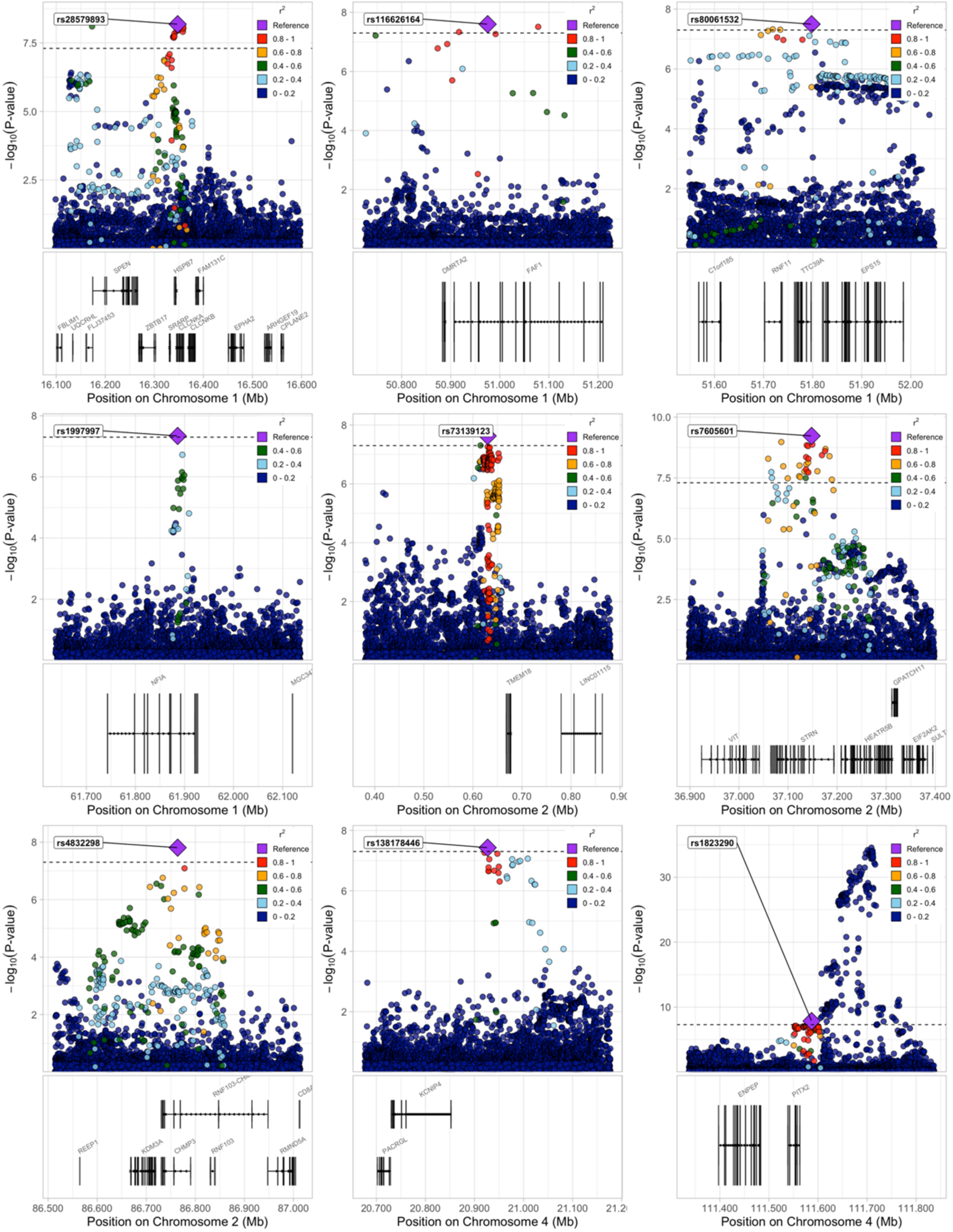

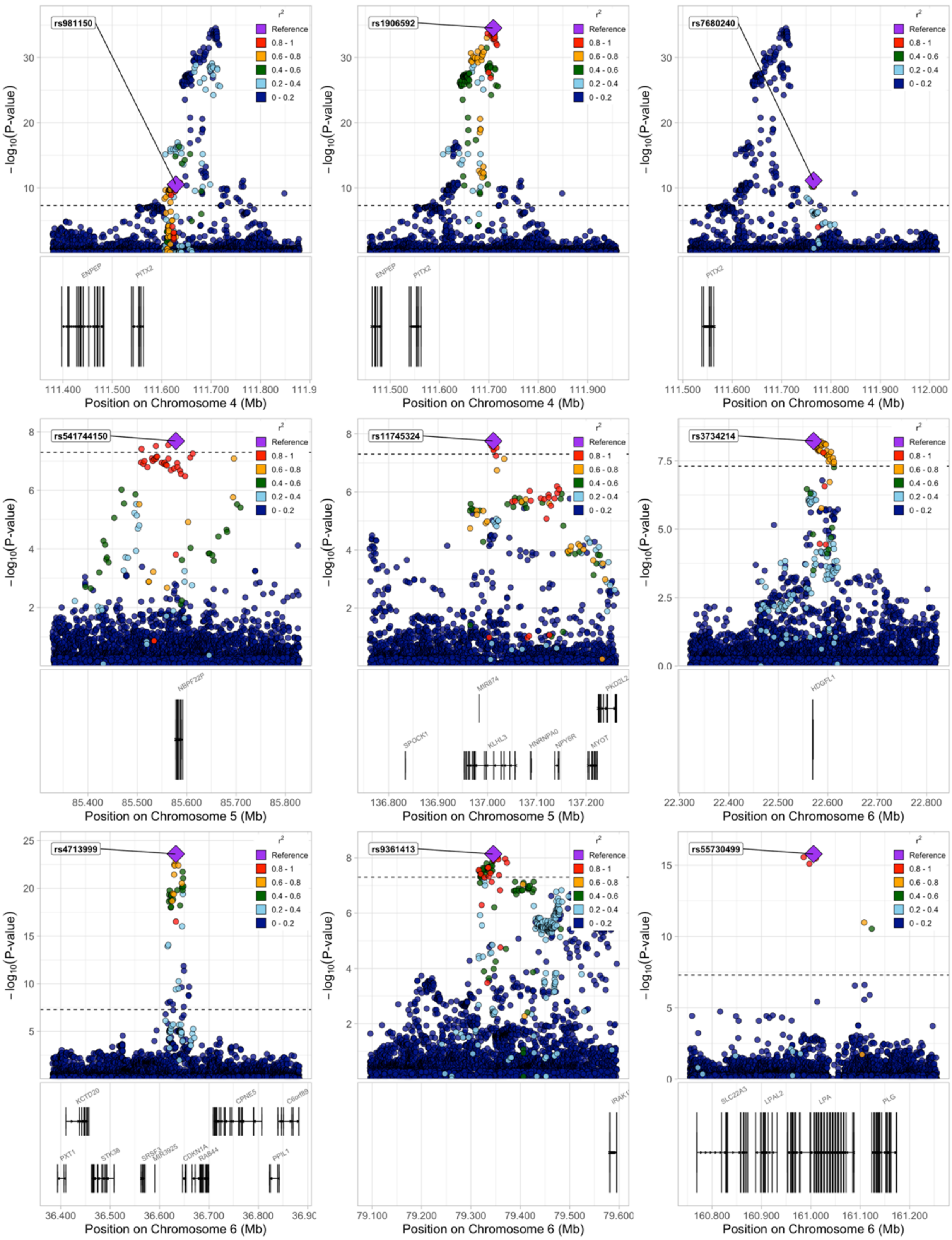
rs981150
rs1906592
rs7680240
rs541744150
rs11745324
rs3734214
rs4713999
rs9361413
rs55730499
r2
Reference
0.8 - 1
0.6 - 0.8
0.4 - 0.6
0.2 - 0.4
0 - 0.2
-log10(P-value)
ENPEP
PITX2
NBPF22P
MIR874
PKD2L2
SPOCK1
KLHL3
HNRNPA0
NPY6R
MYOT
HDGFL1
KCTD20
CPNE5
C6orf89
PXT1
STK38
SRSF3
MIR3925
CDKN1A
RAB44
PPIL1
IRAK1
SLC22A3
LPAL2
LPA
PLG
Position on Chromosome 4 (Mb)
Position on Chromosome 5 (Mb)
Position on Chromosome 6 (Mb)

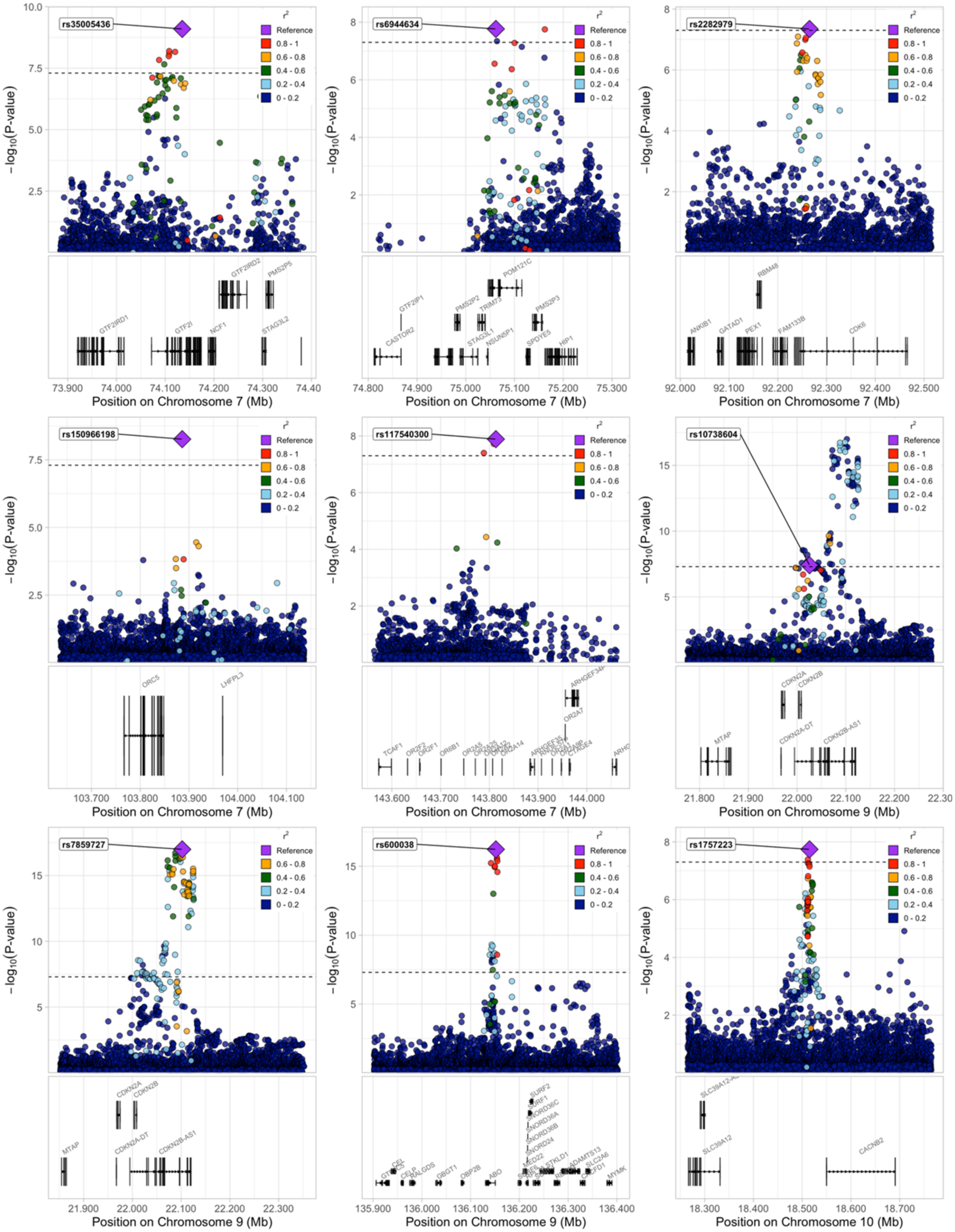

rs35005436
rs6944634
rs2282979
rs150966198
rs117540300
rs10738604
rs7859727
rs600038
rs1757223
r²
Reference
0.8 - 1
0.6 - 0.8
0.4 - 0.6
0.2 - 0.4
0 - 0.2
-log10(P-value)
Position on Chromosome 7 (Mb)
Position on Chromosome 9 (Mb)
Position on Chromosome 10 (Mb)
GTF2IRD2
PMS2P5
GTF2IRD1
GTF2I
NCF1
STAG3L2
POM121C
GTF2IP1
PMS2P2
TRIM73
PMS2P3
CASTOR2
STAG3L1
NSUN5P1
SPDYE5
HIP1
RBM48
ANKIB1
GATAD1
PEX1
FAM133B
CDK6
ORC5
LHFPL3
ARHGEF34P
OR2A7
TCAF1
OR2F2
OR2F1
OR6B1
CTAGE4
CDKN2A
CDKN2B
MTAP
CDKN2A-DT
CDKN2B-AS1
SURF2
SURF1
SNORD36C
SNORD36A
SNORD36B
SNORD24
CEL
CELP
RALGDS
GBGT1
OBP2B
ABO
MED22
ADAMTS13
SLC2A6
MYMK
SLC39A12-AS1
SLC39A12
CACNB2

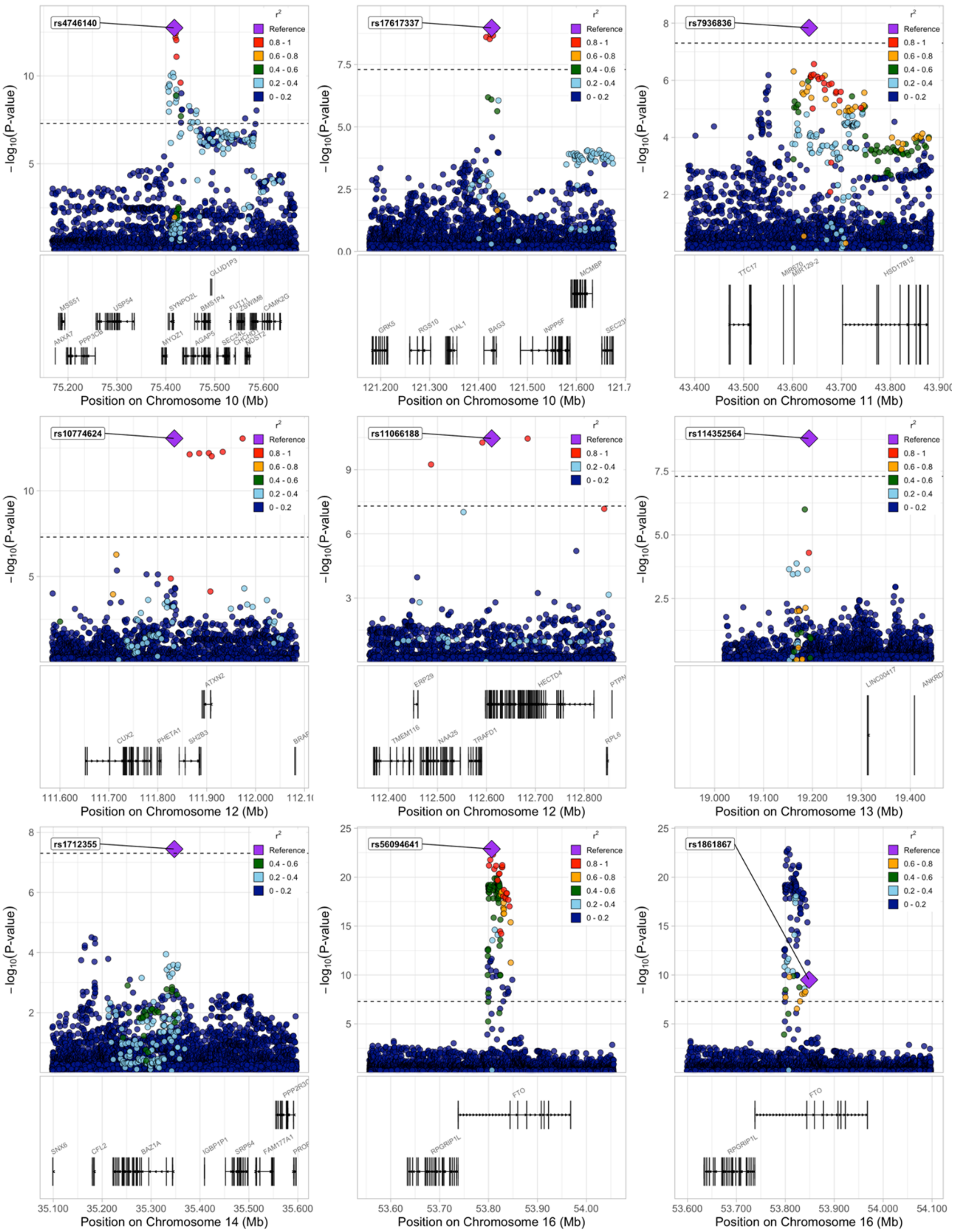
rs4746140
rs17617337
rs7936836
rs10774624
rs11066188
rs114352564
rs1712355
rs56094641
rs1861867
r2
Reference
0.8 - 1
0.6 - 0.8
0.4 - 0.6
0.2 - 0.4
0 - 0.2
-log10(P-value)
Position on Chromosome 10 (Mb)
Position on Chromosome 11 (Mb)
Position on Chromosome 12 (Mb)
Position on Chromosome 13 (Mb)
Position on Chromosome 14 (Mb)
Position on Chromosome 16 (Mb)
MSS51
USP54
SYNPO2L
GLUD1P3
BMS1P4
FUT11
ZSWIM8
CAMK2G
ANXA7
PPP3CB
MYOZ1
AGAP5
NDST2
MCMBP
GRK5
RGS10
TIAL1
BAG3
INPP5F
TTC17
MIR129-2
HSD17B12
ATXN2
CUX2
PHETA1
SH2B3
ERP29
HECTD4
TMEM116
NAA25
TRAFD1
RPL6
LINC00417
SNX6
CFL2
BAZ1A
SRP54
FAM177A1
FTO
RPGRIP1L

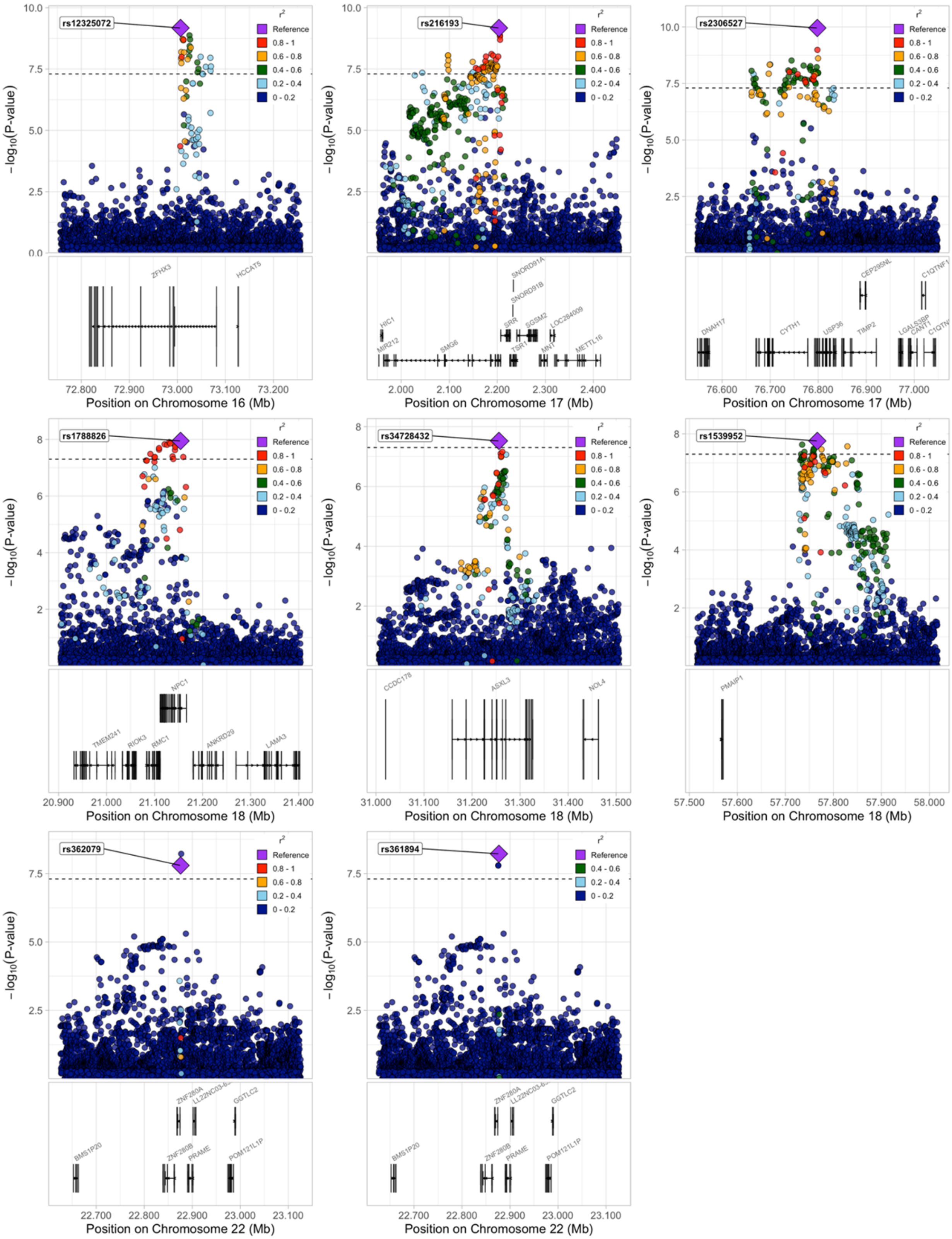

Regional association plots for independent genetic variants associated with HF at $p < 5x10^{-8}$ in the all-cause HF meta-analysis, estimated using fixed-effects with inverse variance weighting. Points are colored by linkage disequilibrium with the index variant, estimated from the 1000 Genomes Phase 3 reference panel.

Supplemental Figure 4: Manhattan Plot of NGWAMA of HF and Cardiac MRI Traits

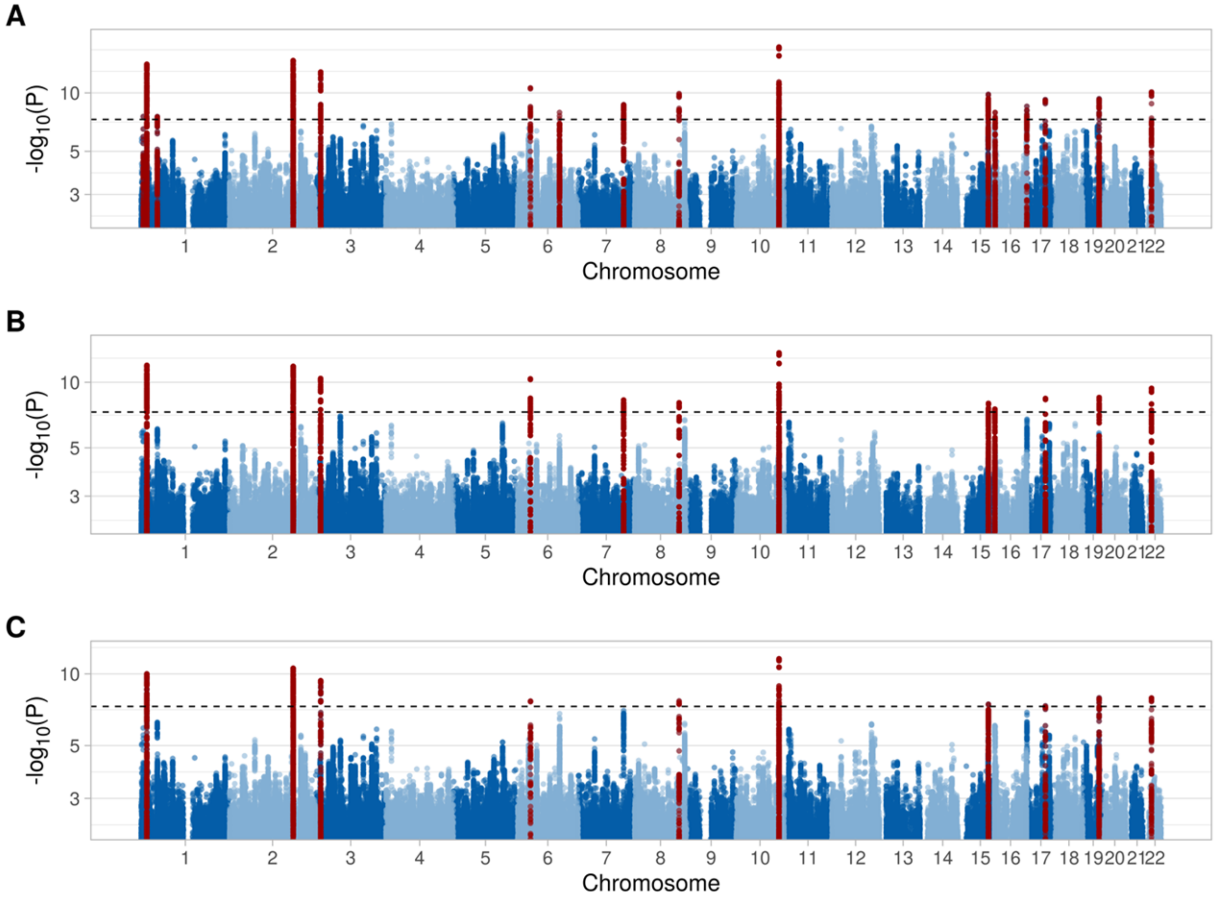

Manhattan Plots of NGWAMA multi-trait GWAS of heart failure and cardiac imaging traits. A) Model including HF and cardiac MRI traits unindexed for body surface area (LVEDV, LVESV, LVEF). B) Model including HF and cardiac MRI traits indexed for body surface area (LVEDVi, LVESVi, LVEF). C) Model including HF and both indexed and unindexed MRI traits (LVEDV, LVEDVi, LVESV, LVESVi, LVEF).

Supplemental Figure 5: Manhattan Plot of MTAG of HF and Cardiac MRI Traits

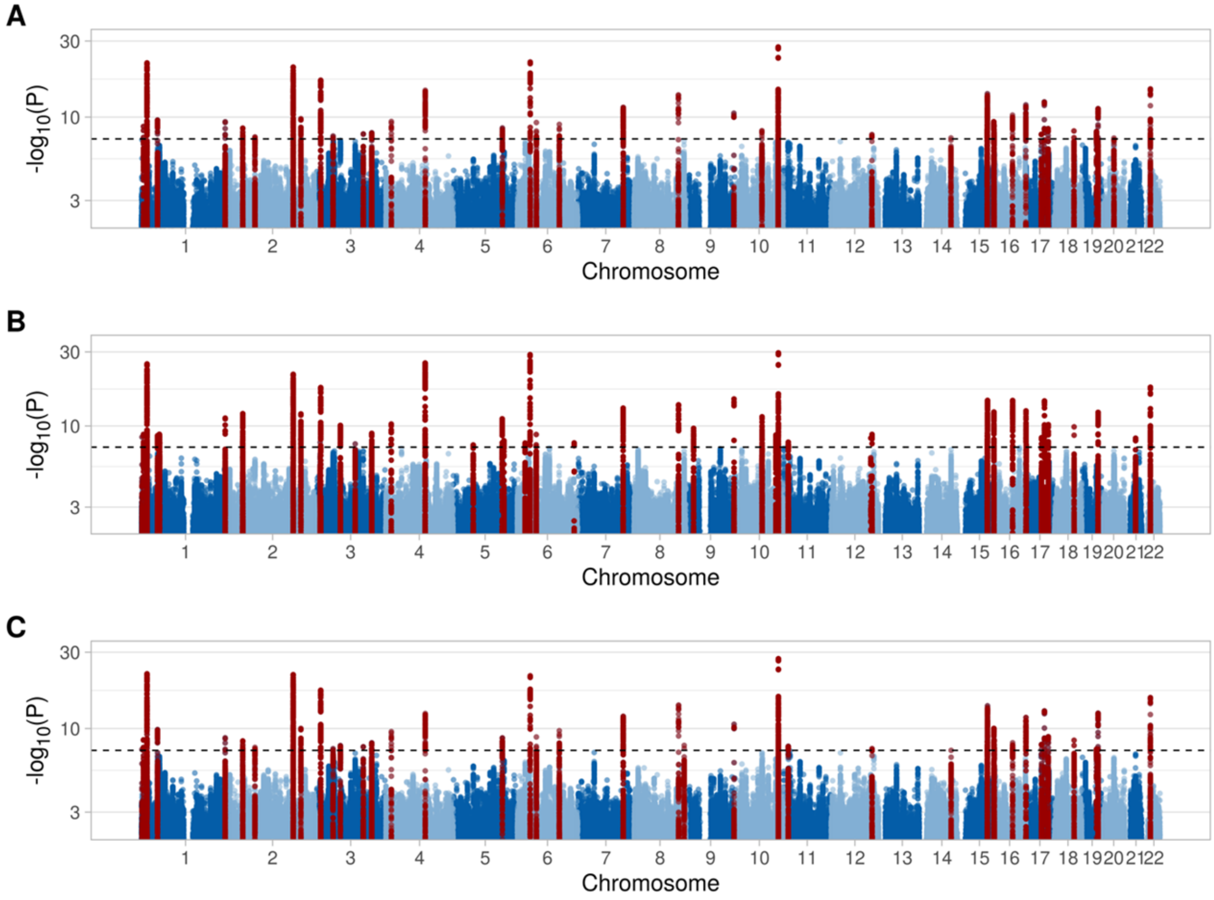

Manhattan Plots of MTAG multi-trait GWAS of heart failure and cardiac imaging traits. A) Model including HF and cardiac MRI traits unindexed for body surface area (LVEDV, LVESV, LVEF). B) Model including HF and cardiac MRI traits indexed for body surface area (LVEDVi, LVESVi, LVEF). C) Model including HF and both indexed and unindexed MRI traits (LVEDV, LVEDVi, LVESV, LVESVi, LVEF).

Supplemental Figure 6: Manhattan Plot of Common Factor GWAS of HF and Cardiac MRI Traits

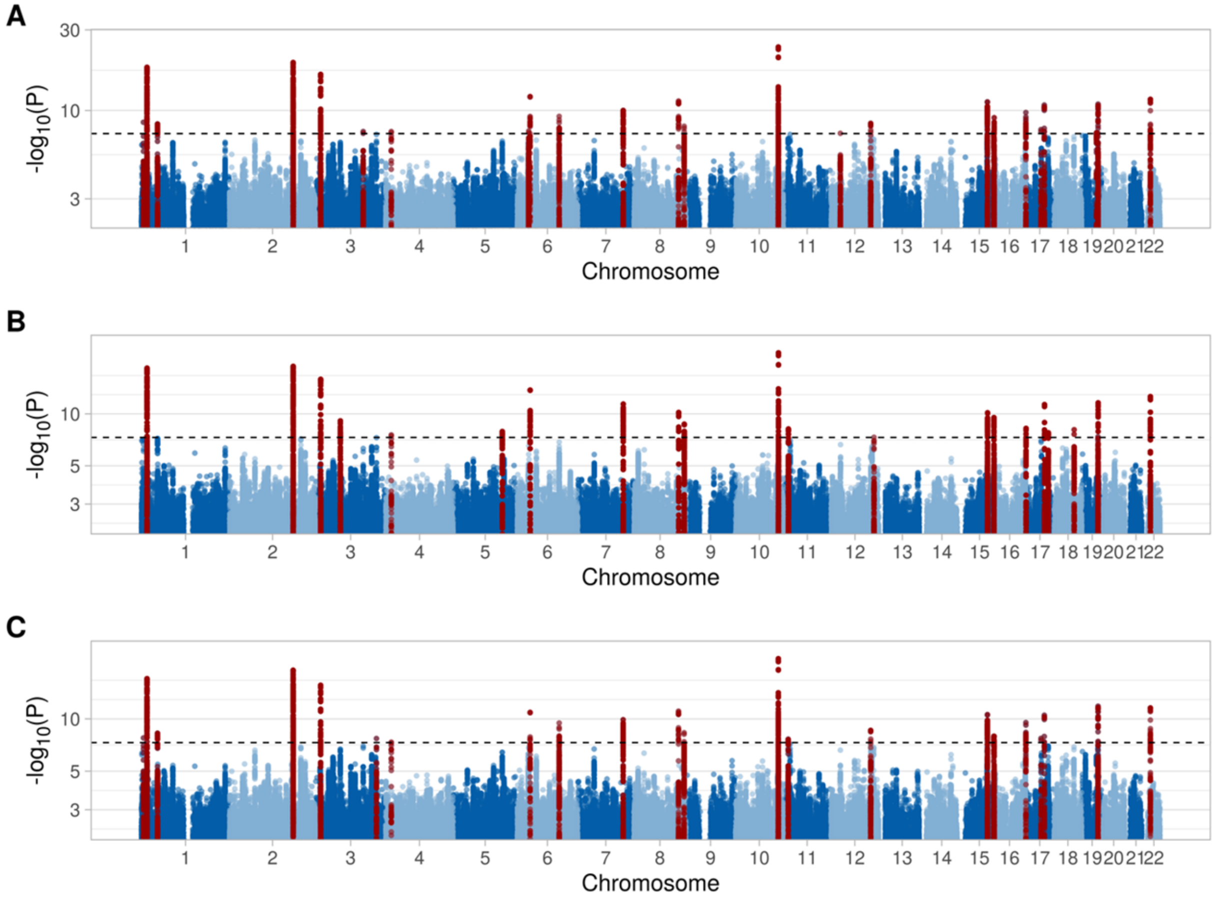

Manhattan Plots of common factor multi-trait GWAS of heart failure and cardiac imaging traits. A) Model including HF and cardiac MRI traits unindexed for body surface area (LVEDV, LVESV, LVEF). B) Model including HF and cardiac MRI traits indexed for body surface area (LVEDVi, LVESVi, LVEF). C) Model including HF and both indexed and unindexed MRI traits (LVEDV, LVEDVi, LVESV, LVESVi, LVEF).

Supplemental Figure 7: Tissue and Cell-type Enrichment

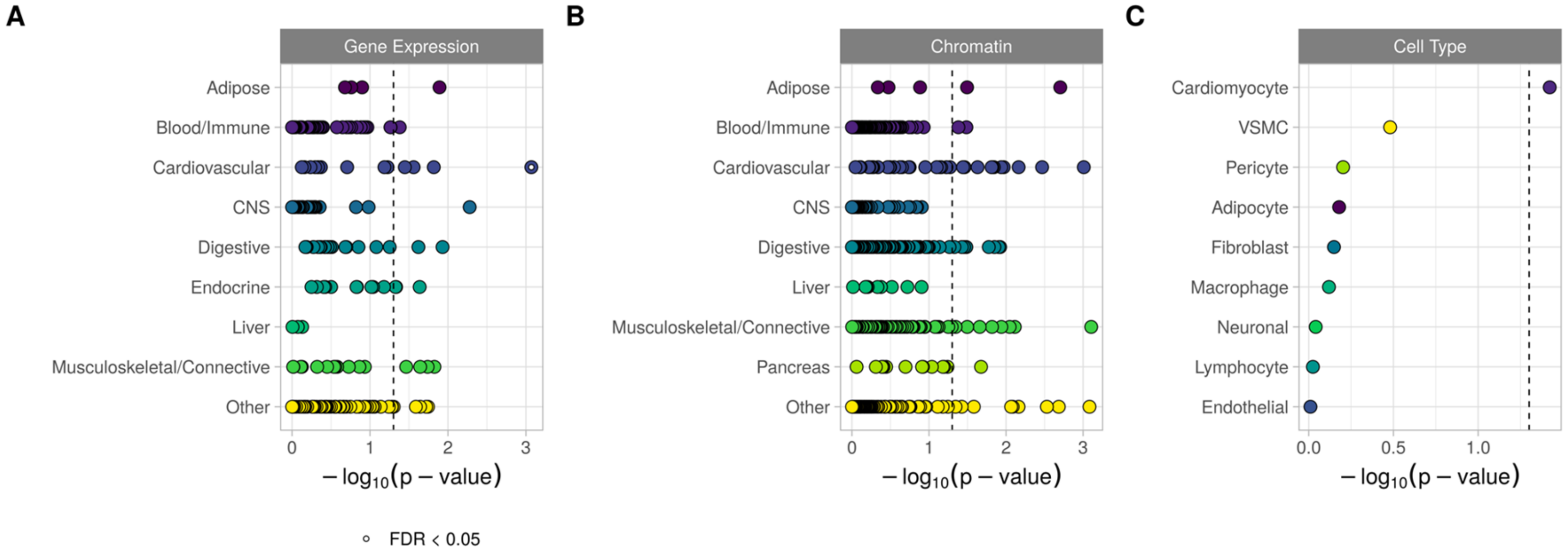

LDSC-SEG was performed to identify tissue and cell-type specific associations with the composite HF endophenotype. A) Association between tissue-specific gene expression (GTEx) and HF. B) Association between tissue-specific chromatin marks (ROADMAP and ENTEX). C) Associations with cardiac-specific cell-types based on differential gene expression. Vertical dashed lines represent nominal significance ($p < 0.05$); FDR significant associations are denoted with white circles.

Supplemental Figure 8: Branch Chain Amino Acid Mendelian Randomization

Exposure	Outcome	n SNP	Method	Beta	95% CI	P
Valine	LVESV-MRI	10	Inverse variance weighted	-0.0108	[-0.063, 0.042]	0.7
Valine	LVEDV-MRI	10	Inverse variance weighted	-0.000399	[-0.063, 0.062]	1
Leucine	LVESV-MRI	5	Inverse variance weighted	-0.131	[-0.24, -0.026]	0.01
Leucine	LVEDV-MRI	5	Inverse variance weighted	-0.137	[-0.25, -0.022]	0.02
Isoleucine	LVESV-MRI	3	Inverse variance weighted	-0.217	[-0.33, -0.11]	1×10^{-4}
Isoleucine	LVEDV-MRI	3	Inverse variance weighted	-0.276	[-0.38, -0.17]	3×10^{-7}

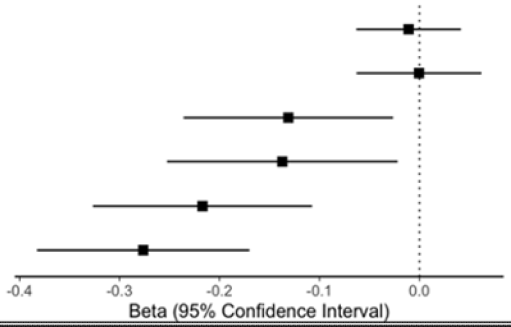

Exposure	Outcome	n SNP	Method	Beta	95% CI	P
Valine	LVESV-MRI	10	Weighted median	-0.00453	[-0.078, 0.068]	0.9
Valine	LVEDV-MRI	10	Weighted median	0.000424	[-0.073, 0.073]	1
Leucine	LVESV-MRI	5	Weighted median	-0.122	[-0.22, -0.028]	0.01
Leucine	LVEDV-MRI	5	Weighted median	-0.113	[-0.21, -0.019]	0.02
Isoleucine	LVESV-MRI	3	Weighted median	-0.21	[-0.33, -0.086]	9×10^{-4}
Isoleucine	LVEDV-MRI	3	Weighted median	-0.273	[-0.4, -0.14]	4×10^{-5}

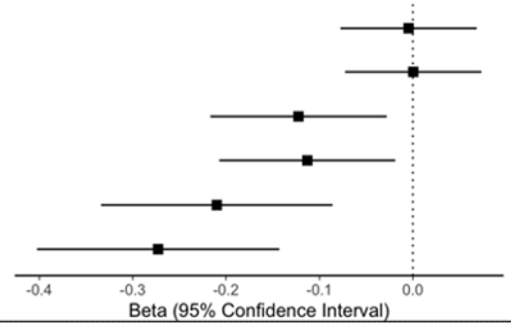

Mendelian randomization was performed to identify whether circulating branch chain amino acid levels were associated with cardiac MRI traits, given colocalization between BCKDHA and both $LVESV_{MRI}$ and $LVEDV_{MRI}$. Presented here are the results of inverse variance weighted and weighted-median MR, which makes different assumptions about the presence of pleiotropy. This method remains robust when up to 50% of the weight of the genetic instrument is derived from invalid instruments. No adjustments were made for multiple comparisons.

Supplemental Figure 9: Summary of Prioritized Genes

A

B

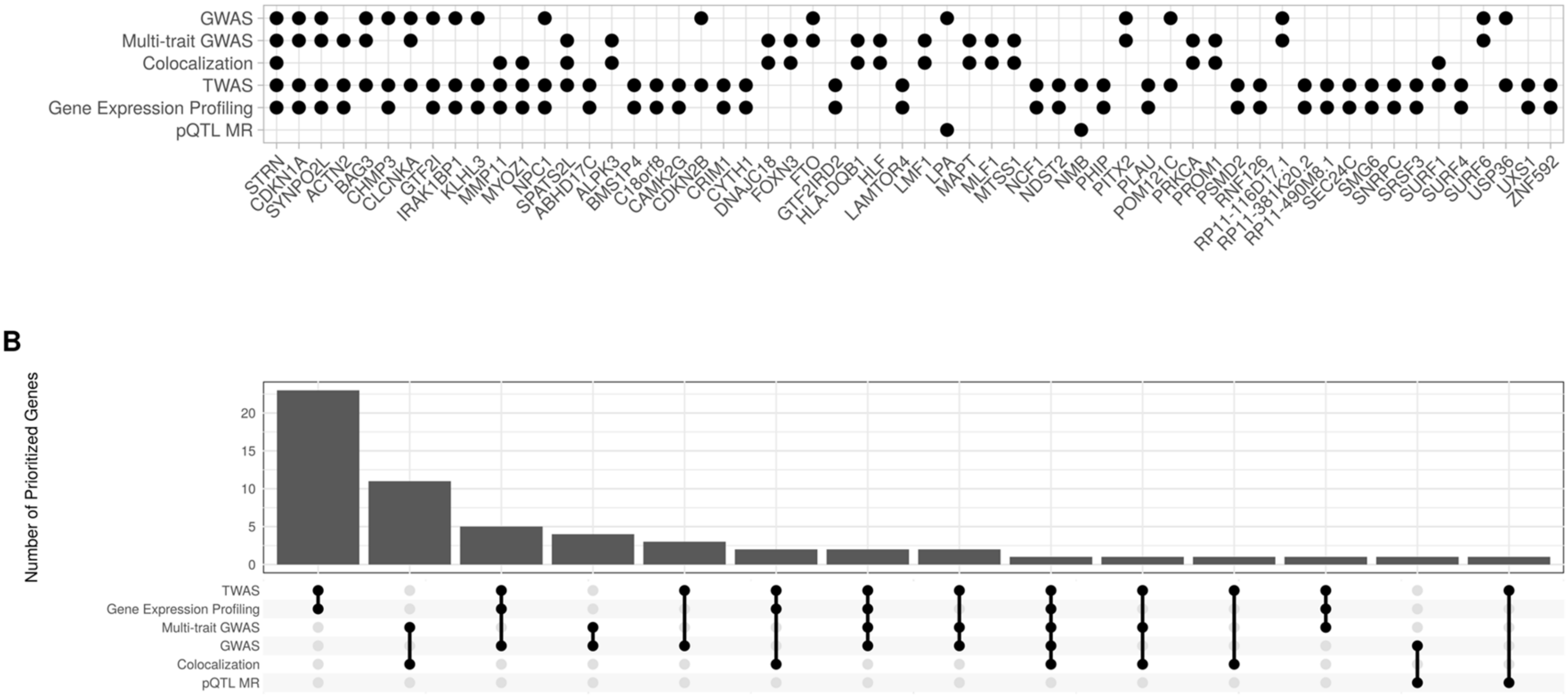

Summary of genes prioritized by two or more different analyses. A) Each gene along the x-axis represents either the nearest gene (prioritized in the GWAS analyses), or specific gene prioritized in the colocalization, TWAS, gene expression profiling, and pQTL MR analyses. Each dot represents a significant gene-analysis pair. B) Summary of concordance in gene prioritization across different methods

SUPPLEMENTAL AUTHORS

Regeneron Genetics Center Banner Author List and Contribution Statements

RGC Management and Leadership Team

Goncalo Abecasis, D.Phil. , Aris Baras, M.D. , Michael Cantor, M.D. , Giovanni Coppola, M.D. , Andrew Deubler , Aris Economides, Ph.D. , Katia Karalis, Ph.D. , Luca A. Lotta, M.D., Ph.D. , John D. Overton, Ph.D. , Jeffrey G. Reid, Ph.D. , Katherine Siminovitch, M.D. , Alan Shuldiner, M.D.

Sequencing and Lab Operations

Christina Beechert , Caitlin Forsythe, M.S. , Erin D. Fuller , Zhenhua Gu, M.S. , Michael Lattari , Alexander Lopez, M.S., John D. Overton, Ph.D. , Maria Sotiropoulos Padilla, M.S. , Manasi Pradhan, M.S. , Kia Manoochehri, B.S. , Thomas D. Schleicher, M.S. , Louis Widom , Sarah E. Wolf, M.S. , Ricardo H. Ulloa, B.S.

Clinical Informatics

Amelia Averitt, Ph.D. , Nilanjana Banerjee, Ph.D. , Michael Cantor, M.D. , Dadong Li, Ph.D. , Sameer Malhotra, M.D. , Deepika Sharma, MHI , Jeffrey Staples , Ph.D.

Genome Informatics

Xiaodong Bai, Ph.D. , Suganthi Balasubramanian, Ph.D. , Suying Bao, Ph.D. , Boris Boutkov, Ph.D. , Siying Chen, Ph.D. , Gisu Eom, B.S. , Lukas Habegger, Ph.D. , Alicia Hawes, B.S. , Shareef Khalid , Olga Krasheninina, M.S. , Rouel Lanche, B.S. , Adam J. Mansfield, B.A. , Evan K. Maxwell, Ph.D. , George Mitra, B.A. , Mona Nafde, M.S. , Sean O'Keeffe, Ph.D. , Max Orelus, B.B.A. , Razvan Panea, Ph.D. , Tommy Polanco, B.A. , Ayesha Rasool, M.S. , Jeffrey G. Reid, Ph.D. , William Salerno, Ph.D. , Jeffrey C. Staples, Ph.D. , Kathie Sun, Ph.D. , Jiwen Xin, Ph.D.

Analytical Genomics and Data Science

Goncalo Abecasis, D.Phil. , Joshua Backman, Ph.D. , Amy Damask, Ph.D. , Lee Dobbyn, Ph.D. , Manuel Allen Revez Ferreira, Ph.D. , Arkopravo Ghosh, M.S. , Christopher Gillies, Ph.D. , Lauren Gurski, B.S. , Eric Jorgenson, Ph.D. , Hyun Min Kang, Ph.D. , Michael Kessler, Ph.D. , Jack Kosmicki, Ph.D. , Alexander Li , Ph.D. , Nan Lin, Ph.D. , Daren Liu, M.S. , Adam Locke, Ph.D. , Jonathan Marchini, Ph.D. , Anthony Marcketta, M.S. , Joelle Mbatchou, Ph.D. , Arden Moscati, Ph.D. , Charles Paulding, Ph.D. , Carlo Sidore, Ph.D. , Eli Stahl, Ph.D. , Kyoko Watanabe, Ph.D. , Bin Ye, Ph.D. , Blair Zhang, Ph.D. , Andrey Ziyatdinov, Ph.D.

Therapeutic Genetics

Luca A. Lotta, M.D., Ph.D., George Hindy, M.D., Ph.D., Niek Verweij, Ph.D., Jonas B. Nielsen, M.D., Ph.D., Tanima De, Ph.D.

Research Program Management & Strategic Initiatives

Marcus B. Jones, Ph.D. , Michelle G. LeBlanc, Ph.D., Jason Mighty, Ph.D. , Lyndon J. Mitnaul, Ph.D.

SUPPLEMENTAL REFERENCES

1. Shah, S. *et al.* Genome-wide association and Mendelian randomisation analysis provide insights into the pathogenesis of heart failure. *Nat. Commun.* **11**, 1–12 (2020).
2. Winkler, T. W. *et al.* Quality control and conduct of genome-wide association meta-analyses. *Nat. Protoc.* **9**, 1192–1212 (2014).
3. Denny, J. C. *et al.* Systematic comparison of phenome-wide association study of electronic medical record data and genome-wide association study data. *Nat. Biotechnol.* **31**, 1102–1111 (2013).
4. Purcell, S. *et al.* PLINK: A tool set for whole-genome association and population-based linkage analyses. *Am. J. Hum. Genet.* **81**, 559–575 (2007).
5. Chang, C. C. *et al.* Second-generation PLINK: Rising to the challenge of larger and richer datasets. *GigaScience* **4**, (2015).
6. Das, S. *et al.* Next-generation genotype imputation service and methods. *Nat. Genet.* **48**, 1284–1287 (2016).
7. Bioconductor Package. *liftOver*. (Bioconductor). doi:10.18129/B9.BIOC.LIFTOVER.
8. Stanaway, I. B. *et al.* The eMERGE genotype set of 83,717 subjects imputed to 40 million variants genome wide and association with the herpes zoster medical record phenotype. *Genet. Epidemiol.* **43**, 63–81 (2019).
9. Manichaikul, A. *et al.* Robust relationship inference in genome-wide association studies. *Bioinformatics* **26**, 2867–2873 (2010).
10. Oetjens, M. T., Kelly, M. A., Sturm, A. C., Martin, C. L. & Ledbetter, D. H. Quantifying the polygenic contribution to variable expressivity in eleven rare genetic disorders. *Nat. Commun.* **10**, 4897 (2019).
11. Zhou, W. *et al.* Global Biobank Meta-analysis Initiative: powering genetic discovery across human diseases. 2021.11.19.21266436 (2021) doi:10.1101/2021.11.19.21266436.
12. Zhou, W. *et al.* Efficiently controlling for case-control imbalance and sample relatedness in large-scale genetic association studies. *Nat. Genet.* **50**, 1335–1341 (2018).
13. Mbatchou, J. *et al.* Computationally efficient whole-genome regression for quantitative and binary traits. *Nat. Genet.* 1–7 (2021) doi:10.1038/s41588-021-00870-7.
14. Loh, P.-R., Palamara, P. F. & Price, A. L. Fast and accurate long-range phasing in a UK Biobank cohort. *Nat. Genet.* **48**, 811–816 (2016).
15. Browning, B. L. & Browning, S. R. Genotype Imputation with Millions of Reference Samples. *Am. J. Hum. Genet.* **98**, 116–126 (2016).
16. Gaziano, J. M. *et al.* Million Veteran Program: A mega-biobank to study genetic influences on health and disease. *J. Clin. Epidemiol.* **70**, 214–223 (2016).

17. Joseph, J. *et al.* Genetic Architecture of Heart Failure with Preserved versus Reduced Ejection Fraction. 2021.12.01.21266829 (2021) doi:10.1101/2021.12.01.21266829.

18. Hunter-Zinck, H. *et al.* Genotyping Array Design and Data Quality Control in the Million Veteran Program. *Am. J. Hum. Genet.* **106**, 535–548 (2020).

19. Fang, H. *et al.* Harmonizing Genetic Ancestry and Self-identified Race/Ethnicity in Genome-wide Association Studies. *Am. J. Hum. Genet.* **105**, 763–772 (2019).

20. Karlson, E. W., Boutin, N. T., Hoffnagle, A. G. & Allen, N. L. Building the Partners HealthCare Biobank at Partners Personalized Medicine: Informed Consent, Return of Research Results, Recruitment Lessons and Operational Considerations. *J. Pers. Med.* **6**, E2 (2016).

21. Grotzinger, A. D. *et al.* Genetic architecture of 11 major psychiatric disorders at biobehavioral, functional genomic and molecular genetic levels of analysis. *Nat. Genet.* 1–12 (2022) doi:10.1038/s41588-022-01057-4.

22. Turley, P. *et al.* Multi-trait analysis of genome-wide association summary statistics using MTAG. *Nat. Genet.* **50**, 229–237 (2018).

23. Grotzinger, A. D. *et al.* Genomic structural equation modelling provides insights into the multivariate genetic architecture of complex traits. *Nat. Hum. Behav.* **3**, 513–525 (2019).

24. Baselmans, B. M. L. *et al.* Multivariate genome-wide analyses of the well-being spectrum. *Nat. Genet.* **51**, 445–451 (2019).

25. Privé, F. *Using the UK Biobank as a global reference of worldwide populations: application to measuring ancestry diversity from GWAS summary statistics*. 2021.10.27.466078 https://www.biorxiv.org/content/10.1101/2021.10.27.466078v2 (2021) doi:10.1101/2021.10.27.466078.

26. Privé, F., Aschard, H., Ziyatdinov, A. & Blum, M. G. B. Efficient analysis of large-scale genome-wide data with two R packages: bigstatsr and bigsnpr. *Bioinforma. Oxf. Engl.* **34**, 2781–2787 (2018).
