# Supplementary material for: Genome-wide association and multi-trait analyses characterize the common genetic architecture of heart failure
Source: Nat Commun. 2022 Nov 14;13:6914. doi: 10.1038/s41467-022-34216-6 (PMC9663424; doi:10.1038/s41467-022-34216-6)
Supplement: Supplementary file 2 — Description of Additional Supplementary Files [file 41467_2022_34216_MOESM2_ESM.pdf]

**Description of Additional Supplementary Files**

File Name: **Supplementary Data 1**
Description: **Cohorts**. Sample sizes of contributing cohorts.

File Name: **Supplementary Data 2**
Description: **HF Loci**. 47 loci (r2 < 0.1, distance = 500kb) that reached the genome-wide significance (GWS) threshold (p < 5 x 10-8) for association with HF after fixed-effects meta analysis and present in at least 2 studies; loci were annoted with VEP to identify the nearest gene.

File Name: **Supplementary Data 3**
Description: **HF Replication**. Replication of the 47 loci identified in the discovery phase was performed in the VA Million Veteran Program and Mass General Brigham Biobank. MVP and MGB summary statistics were available at 44/47 loci, and were combined using fixed-effects meta-analysis. The exact binomial test was performed to evaluate for concordant direction of effect, with p < 0.05 considered significant. In a combined discovery + replication meta-analysis, 39/44 loci reached the genome-wide significance threshold (p < 5 x 10-8).

File Name: **Supplementary Data 4**
Description: **HF pheWAS**. Associations between lead HF loci and traits estimated by GWAS in the MRC-IEU OpenGWAS Project database. No adjustment was made for multiple comparisons.

File Name: **Supplementary Data 5**
Description: **HF Risk Factors**. Associations between lead HF loci and cardiometabolic risk factors for HF estimated by GWAS. ".exposure" columns refer to HF, while ".outcome" columns refer to each risk factor. Unadjusted p-values are listed.

File Name: **Supplementary Data 6**
Description: **LD Score Regression.** Cross-trait LD Score Regression results for genetic correlation analysis of HF and cardiac imaging traits. Unadjusted p-values are listed. Correlations meeting a Bonferroni adjustment for mulitiple testing are noted in Figure 4A.

File Name: **Supplementary Data 7**
Description: **Multi-trait GWAS Loci.** GenomicSEM output for 61 lead loci identified in multi-trait GWAS of HF and cardiac MRI traits; annotation was performed with VEP to identify the nearest genet at each locus.

File Name: **Supplementary Data 8**
Description: **Multi-trait GWAS GWS variants.** GenomicSEM output for all genome-wide significant (p < 5e-8) variants in each multi-trait (MTAG, N-GWAMA, common factor) GWAS of HF and cardiac MRI traits across 3 models (HF + MRI parameters unindexed for body surface area; HF + MRI parameters indexed for body surface area; and HF + both indexed and unindexed MRI parameters); annotation was performed with VEP to identify the nearest genet at each locus. No adjustment for multiple testing was performed.

File Name: **Supplementary Data 9**
Description: **Multi-trait colocalization**. Results of HyPrColoc, which used colocalization to evaluated shared risk variants across HF and cardiac MRI traits at each lead locus from the multi-trait GWAS. Loci with evidence of colocalization (PrPa > 0.25) are presented.

File Name: **Supplementary Data 10**
Description: **Multi-trait pheWAS**. The lead SNP at novel locus in the multi-trait GWAS were queried in the Open GWAS Project database of 34,513 GWAS to identify other traits associated with these loci. These results were reviewed qualitatively and no adjustment was made for multiple comparisons.

File Name: **Supplementary Data 11**
Description: **LDSC-SEG results**. Three enrichment analyses using LDSC-SEG were performed to test associations with NGWAMA loci and gene expression, chromatin marks, and cardiac-specific cell-types. FDR was applied across each analysis to account for multiple comparisons.

File Name: **Supplementary Data 12**
Description: **HF-MAGNet colocalization.** Results of colocalization between genetic variants associated with heart failure and gene expression from healthy/failing hearts from the MAGNet consortium.

File Name: **Supplementary Data 13**
Description: **eQTL TWAS Results**. Highly prioritized gene-tissue pairs from TWAS (S-prediXcan) of gene expression in metabolically active tissues and HF (gene-tissue pairs with Bonferroni $p < 0.05$).

File Name: **Supplementary Data 14**
Description: **sQTL TWAS Results**. Highly prioritized gene-tissue pairs from TWAS (S-prediXcan) of gene splicing events in metabolically active tissues and HF (gene-splicing pairs with Bonferroni $p < 0.05$).

File Name: **Supplementary Data 15**
Description: **Gene Expression Profiling Results.** Differential gene expression for the 66 genes prioritized in the TWAS analysis was assessed in MAGnet. Bonferroni adjustment was performed to account for multiple comparisons.

File Name: **Supplementary Data 16**
Description: **TWAS Cellular Components.** Results of enrichment testing of TWAS prioritized genes using GO Cellular components with ShinyGO v0.75.

File Name: **Supplementary Data 17**
Description: **TWAS Biologial Processes**. Results of enrichment testing of TWAS prioritized genes using GO Biological processes with ShinyGO v0.75.

File Name: **Supplementary Data 18**
Description: **pQTL Mendelian Randomization Results**. Mendelian randomization was performed to evaluate the association of circulating proteins with risk of heart failure and cardiac imaging parameters. Wald ratio MR was performed when a single genetic instrument was available, and inverse-variance weighting was used to combine effects when multiple genetic instruments were available. The false discovery rate was controlled at 5% to account for multiple comparisons.
