# Supplementary material for: Genome-wide association and multi-trait analyses characterize the common genetic architecture of heart failure
Source: Nat Commun. 2022 Nov 14;13:6914. doi: 10.1038/s41467-022-34216-6 (PMC9663424; doi:10.1038/s41467-022-34216-6)
Supplement: Supplementary file 4 — Reporting Summary [file 41467_2022_34216_MOESM4_ESM.pdf]

Corresponding author(s): Scott Damrauer
Last updated by author(s): October 10, 2022

Reporting Summary

Nature Portfolio wishes to improve the reproducibility of the work that we publish. This form provides structure for consistency and transparency in reporting. For further information on Nature Portfolio policies, see our Editorial Policies and the Editorial Policy Checklist.

Statistics

For all statistical analyses, confirm that the following items are present in the figure legend, table legend, main text, or Methods section.

n/a	Confirmed	
☐	☒	The exact sample size (*n*) for each experimental group/condition, given as a discrete number and unit of measurement
☐	☒	A statement on whether measurements were taken from distinct samples or whether the same sample was measured repeatedly
☐	☒	The statistical test(s) used AND whether they are one- or two-sided<br>*Only common tests should be described solely by name; describe more complex techniques in the Methods section.*
☐	☒	A description of all covariates tested
☐	☒	A description of any assumptions or corrections, such as tests of normality and adjustment for multiple comparisons
☐	☒	A full description of the statistical parameters including central tendency (e.g. means) or other basic estimates (e.g. regression coefficient) AND variation (e.g. standard deviation) or associated estimates of uncertainty (e.g. confidence intervals)
☐	☒	For null hypothesis testing, the test statistic (e.g. *F*, *t*, *r*) with confidence intervals, effect sizes, degrees of freedom and *P* value noted<br>*Give P values as exact values whenever suitable.*
☐	☒	For Bayesian analysis, information on the choice of priors and Markov chain Monte Carlo settings
☒	☐	For hierarchical and complex designs, identification of the appropriate level for tests and full reporting of outcomes
☒	☐	Estimates of effect sizes (e.g. Cohen's *d*, Pearson's *r*), indicating how they were calculated

Our web collection on statistics for biologists contains articles on many of the points above.

Software and code

Policy information about availability of computer code

Data collection	No software was used for data collection
Data analysis	GenomicSEM (v0.0.5) https://github.com/GenomicSEM/GenomicSEM<br>GWASinspector (v1.5.7.2) http://gwasinspector.com/<br>HyPrColoc (v1.0) https://github.com/jrs95/hyprcoloc<br>LDSC (v1.0.1) https://github.com/bulik/ldsc<br>METAL (v2020-05-05) https://genome.sph.umich.edu/wiki/METAL<br>R (v4.0.3) (R Foundation for Statistical Computing, Vienna, Austria)<br>PLINK (v1.9) https://www.cog-genomics.org/plink/1.9/<br>Proteome-wide Mendelian Randomization: https://www.epigraphdb.org/pqtl/<br>ShinyGO (v0.75) http://bioinformatics.sdstate.edu/go/<br>S-PrediXcan (v2018-08-29) https://github.com/hakyimlab/MetaXcan<br>TwoSampleMR (v0.5.6) https://github.com/MRCIEU/TwoSampleMR

For manuscripts utilizing custom algorithms or software that are central to the research but not yet described in published literature, software must be made available to editors and reviewers. We strongly encourage code deposition in a community repository (e.g. GitHub). See the Nature Portfolio guidelines for submitting code & software for further information.

Data

Policy information about availability of data

All manuscripts must include a data availability statement. This statement should provide the following information, where applicable:

- Accession codes, unique identifiers, or web links for publicly available datasets
- A description of any restrictions on data availability
- For clinical datasets or third party data, please ensure that the statement adheres to our policy

The data sets generated as part of this study are available from the corresponding author upon reasonable request. The GWAS summary statistics for heart failure (HERMES: http://kp4cd.org/datasets/mi; GBMI: https://www.globalbiobankmeta.org/; FinnGen: https://r5.finngen.fi/pheno/I9_HEARTFAIL_ALLCAUSE), and cardiac MRI (http://kp4cd.org/datasets/mi) traits are publicly available. Cardiac eQTL and RNA expression/sequencing data were provided by the Myocardial Applied Genomics Network (MAGNet; https://www.med.upenn.edu/magnet/). The summary statistics for the GWAS of all-cause heart failure generated in this study have been deposited in the GWAS Catalog database under accession code GCST90162626. The summary statistics for the GWAS of all-cause heart failure and the multi-trait GWAS have also been deposited at Zenodo at http://doi.org/10.5281/zenodo.7181277.

Field-specific reporting

Please select the one below that is the best fit for your research. If you are not sure, read the appropriate sections before making your selection.

☒ Life sciences ☐ Behavioural & social sciences ☐ Ecological, evolutionary & environmental sciences

For a reference copy of the document with all sections, see nature.com/documents/nr-reporting-summary-flat.pdf

Life sciences study design

All studies must disclose on these points even when the disclosure is negative.

Sample size	In our heart failure GWAS meta-analysis we included 115,150 HF cases and 1,550,331 controls of diverse genetic ancestry. We have combined previously unpublished data from the Penn Medicine Biobank, the Geisinger DiscovEHR biobank, Mount Sinai BioMe, and the eMERGE consortium, as well as publicly available summary data from the Global Biobank Meta-analysis Initiative, BioBank Japan, FinnGen, and the HERMES consortium. These represented the largest available datasets for analysis. Given previously reported genetic discovery with smaller sample sizes and the known relationships between sample size and power for genetic discovery, these represented appropriate datasets for analysis.
Data exclusions	In both the heart failure and multivariate GWAS meta-analysis genetic variants were filtered to include include common (MAF > 0.01) variants present in the 1000 Genomes Phase 3 reference panel.
Replication	We sought replication for the univariate heart failure GWAS meta analysis (HERMES + BioBank Japan) from FinnGen release 4 (http://r4.finngen.fi/pheno/I9_HEARTFAIL_ALLCAUSE), which included 17,387 all-cause HF cases and 159,058 controls. These were the only datasets used for replication, as they represent the largest known datasets not included in the discovery analysis.
Randomization	Cases were individuals with heart failure, and controls were disease-free, with specific heart failure phenotyping performed among the original studies based on diagnosis codes. Genetic association tests were adjusted for age, sex, population structure, and other study-specific covariates noted in the original publications. This study was an observational genetic epidemiology study without an interventional arm, and randomization was therefore not relevant.
Blinding	Blinding was not relevant to this study as it made use of previously published/collected GWAS summary statistics. This study was an observational genetic epidemiology study which made use of previously collected datasets. Participants were not allocated to a treatment or intervention.

Reporting for specific materials, systems and methods

We require information from authors about some types of materials, experimental systems and methods used in many studies. Here, indicate whether each material, system or method listed is relevant to your study. If you are not sure if a list item applies to your research, read the appropriate section before selecting a response.

Materials & experimental systems

n/a	Involved in the study
☒	☐ Antibodies
☒	☐ Eukaryotic cell lines
☒	☐ Palaeontology and archaeology
☒	☐ Animals and other organisms
☐	☒ Human research participants
☒	☐ Clinical data
☒	☐ Dual use research of concern

Methods

n/a	Involved in the study
☒	☐ ChIP-seq
☒	☐ Flow cytometry
☒	☐ MRI-based neuroimaging

Human research participants

Policy information about studies involving human research participants

Population characteristics	This project utilized summary genetic data from previously-published/collected studies. Individual-level demographic descriptions are available from the original publications.
Recruitment	Individuals from the HERMES study were participants in either case/control or population-based studies, while participants from BioBank Japan, UK Biobank, FinnGen, Penn Medicine Biobank, eMERGE, BioME, VA Million Veteran Program, and Geisinger were participants of the respective biobanks.
Ethics oversight	The UK Biobank obtained IRB approval from the North West Multi-centre Research Ethics Committee (approval number: 11/NW/0382), and participants provided informed consent. The BioBank Japan Project was approved by the research ethics committees at the Institute of Medical Science, the University of Tokyo, the RIKEN Yokohama Institute, and cooperating hospitals; participants gave written informed consent. FinnGen participants provided informed consent for biobank research, and the Coordinating Ethics Committee of the Hospital District of Helsinki and Uusimaa (HUS) approved the FinnGen Study protocol No. HUS/990/2017. The Penn Medicine BioBank is approved by the University of Pennsylvania, and participants gave written informed consent.

Note that full information on the approval of the study protocol must also be provided in the manuscript.
